# Peat fires contribute disproportionately to Siberian fire carbon emissions

**DOI:** 10.1126/sciadv.adl2368

**Published:** 2026-03-18

**Authors:** Amin Khairoun, Philippe Ciais, Thu Hang Nguyen, Chunjing Qiu, Chengliuhui Fang, Filipe Aires, Sander Veraverbeke, Clement J. F. Delcourt, Bo Zheng, Emilio Chuvieco

**Affiliations:** ^1^ Universidad de Alcalá, Environmental Remote Sensing Research Group, Department of Geology, Geography and the Environment, Alcalá de Henares, Spain.; ^2^ Laboratoire des Sciences du Climat et de l’Environnement, UMR 1572 CEA-CNRS-UVSQ, Université Paris-Saclay, 91191 Gif-sur-Yvette Cedex, France.; ^3^ Research Center for Global Change and Ecological Forecasting, School of Ecological and Environmental Sciences, East China Normal University, Shanghai, China.; ^4^ LIRA, CNRS/Observatoire de Paris/Sorbonne University, Paris, France.; ^5^ Faculty of Science, Vrije Universiteit Amsterdam, Amsterdam, Netherlands.; ^6^ School of Environmental Sciences, University of East Anglia, Norwich, UK.; ^7^ Shenzhen Key Laboratory of Ecological Remediation and Carbon Sequestration, Institute of Environment and Ecology, Tsinghua Shenzhen International Graduate School, Tsinghua University, Shenzhen 518055, China.; ^8^ State Environmental Protection Key Laboratory of Sources and Control of Air Pollution Complex, Beijing 100084, China.

## Abstract

Arctic and boreal fires are critical threats to terrestrial carbon reservoirs, particularly peat fires that trigger long-term irrecoverable carbon losses and permafrost thaw. However, the occurrence of peat fires and their associated carbon emissions remain highly uncertain. 30-meter satellite-derived maps of burned area and peatland coverage reveal that Siberian fires burned over 107 million hectares during the 2001 to 2023 period, with peat fires accounting for up to one-third of this area. These peat fires emitted 1.24 ± 0.06 petagram of carbon, largely exceeding conventional datasets’ estimates. We found that anomalous dry and warm climatic conditions represent the primary driver of extreme peat fire seasons and that overwintering of 2020’s late-season peat fires substantially contributed to extensive fires of 2021. Peat fires, especially those in Arctic regions, exhibit a pronounced sensitivity to extreme weather, posing a critical threat to the stability of permafrost peatlands and their large carbon stocks.

## Teaser

Siberian peatlands are strongly affected by fires in recent years due to extreme drought.

## INTRODUCTION

Northern peatlands represent one of the largest carbon (C) pools in the biosphere and play a critical role in the regulation of the Earth’s climate. Although they represent only 2 to 3% of terrestrial surface, northern peatlands store extensive amounts of soil organic carbon (SOC) (*1*, *2*). These ecosystems hold ~415 ± 150 Pg of C, developed from a slow accumulation of dead organic material under generally saturated conditions. Northern peatlands constitute a persistent long-term sink of atmospheric carbon dioxide (CO_2_) and a notable methane (CH_4_) source due to anoxic decomposition (*3*). With temperatures rising up to four times faster in the Arctic than the global average (*4*, *5*) permafrost thaw in peatlands has accelerated (*6*), leading to an increased decomposition of organic matter and higher release of greenhouse gases (*7*). The persistence of this carbon sink is under growing threat from increasing disturbances, including fire activity, which can rapidly release stored carbon and reinforce climate warming (*8*, *9*).

Northern peatland wildfires have generally been infrequent throughout the Holocene, but the unusually warm and dry conditions experienced in several recent years (i.e., 2019, 2020, and 2021) promoted a substantial surge of fire activity in Siberian peatlands (*10*). In addition to the acceleration of permafrost thaw, these events alter the postfire recovery of permafrost landscapes by promoting the expansion of warmer and deeper active layers of thermokarst bogs (*11*) and lead to a substantial rise of carbon emissions (*12*, *13*). On average, the current coarse-resolution (>250 m) burned area (BA) products estimate ~8 Mha of Arctic-boreal fires per year, representing only 1.6% of the global BA, yet they account for more than 7% of the global carbon emissions from fires (*12*). Most of these fires occur in the northern larch forests and tundra of central and eastern Siberia landscapes predominately associated with carbon-rich peatlands (*10*). Subsequently, more than 70% of boreal fire emissions result from belowground burning of SOC (*14*). Existing coarse-resolution datasets tend to underestimate BA (*15*, *16*), leading to high uncertainties in the assessment of their trends (*17*) and impacts (*16*, *18*), especially in the context of boreal peatlands where rising temperature is assumed to be a major driver of fire severity (e.g., burn depth in organic soil) and frequency (*11*, *19*).

Although most BA products rely on the Moderate Resolution Imaging Spectroradiometer (MODIS) satellite observations, the methodologies used in current fire emission datasets differ with their estimations showing substantial disparities globally, particularly in Arctic-boreal regions. For example, Pan *et al.* (*20*) compared global emissions derived from six fire emission datasets and found that the differences in estimations reach a factor of 3.8 globally and more than 5 in boreal and Arctic Asia (including Siberia). In southern Siberia, large disparities were observed between successive versions of the Global Fire Emissions Database (GFED) products, with the recent GFED5 dataset including more small fires (*21*) producing four times more BA than its predecessor GFED4s (*22*), which is expected to lead to large differences in terms of fire emissions. In general, combining high-resolution BA datasets with finer modeling grids brings substantial improvements in capturing wildfire emissions (*14*, *16*, *23*). On the other hand, process-based model simulations of the Fire Modelling Intercomparison Project (*24*) are not capable of successfully capturing the decadal trends of fire CO_2_ emissions and fire emissions intensity (*17*). Furthermore, the estimation of the fraction of peat fires remains either unavailable or highly uncertain. For instance, the estimation of the fraction of BA that are peat fires in GFED4s is underestimated in boreal and Arctic Asia, while it is absent in process-based models. Several recent works have attempted to map the distribution of northern peatlands (*3*, *25*–*28*). However, these datasets typically provide a coarse representation of Siberian peatland coverage (i.e., spatial resolution ≥ 1 km), which could not be matched with high-resolution BA information to separate peat fires.

The objective of this work is to provide an accurate evaluation of the impacts of wildfires in Siberian peatlands based on high-resolution satellite data. We developed a wall-to-wall characterization of peat fires in Northern Siberia (>58° N) at high spatial resolution using Landsat data for the period 2001 to 2023. BA was mapped at 30-m spatial resolution over the last 23 years and overlaid with a new 90-m peatland coverage map (see Materials and Methods). We analyzed the trend and characteristics of wildfires in peat-dominated Arctic areas and compared them with subarctic boreal forest fires. Then, we used a machine learning model to estimate burn depth and carbon emissions due to fires based on fire characteristics in combination with several categories of other predictors (climate, soil, topography, etc.). Last, we assessed the strength of causal relationships between climate and fire activity to better understand their interactions.

## RESULTS

### Spatiotemporal distribution of fires

**Fig. 1.**
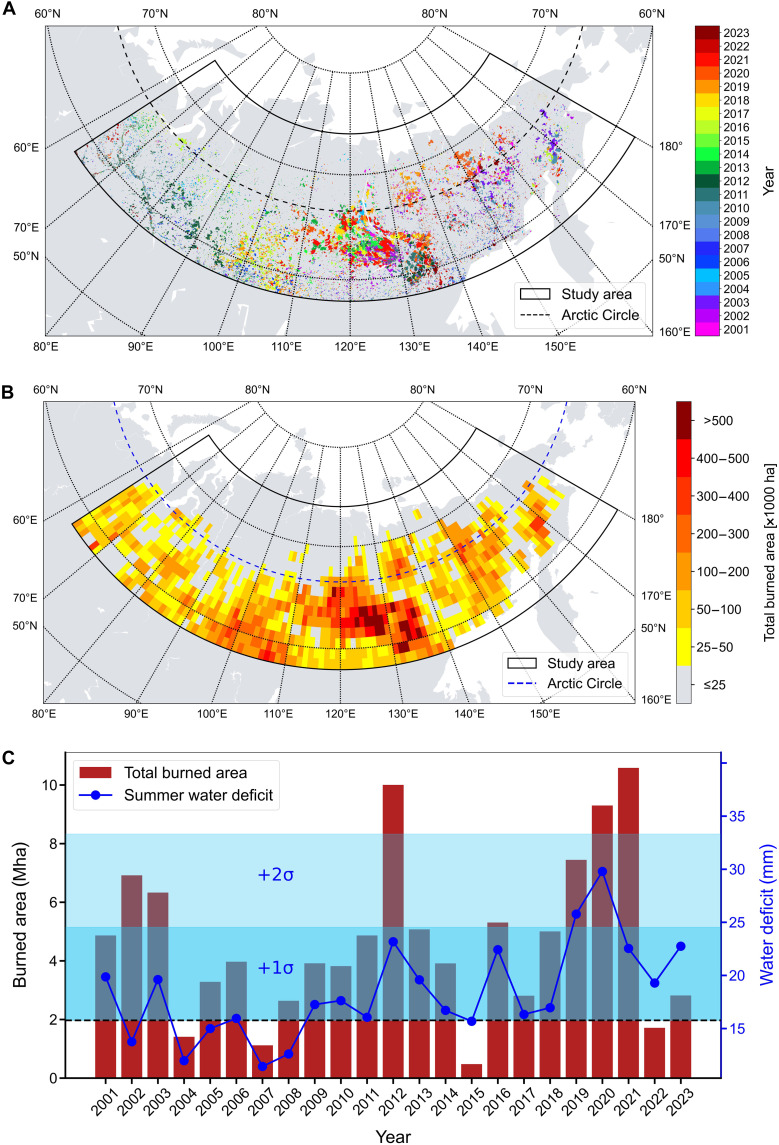
Siberian fire activity during the period 2001 to 2023. (**A**) Distribution of yearly fires. (**B**) Total BA during the period 2001 to 2023 aggregated over 1° by 1° grid cells. (**C**) Total annual BA and climatic water deficit for the period 2001 to 2023. The black dashed line designates the long-term (1958 to 2023) summer mean of climatic water deficit while shading indicates one and two one-sided SDs from this mean.

**Fig. 2.**
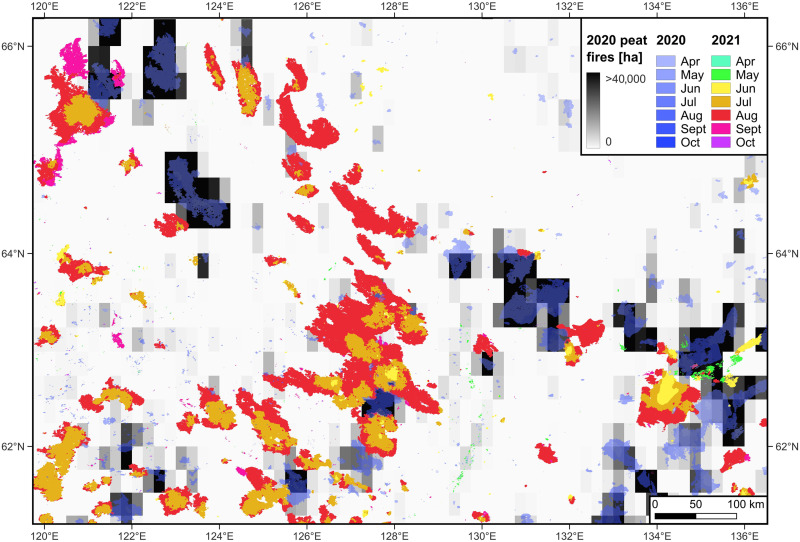
Illustrations of the impact of overwintering fires in the 2020 to 2021 fire seasons. The maps show the reemergence of late 2020 fire season expanding over large fire swaths in 2021, and their corresponding peat fires area reported at 0.25° grids. The largest fire patch of 2021 extends from 124.98°E, 61.88°N to 129.11°E, 63.84°N.

Our study region covers most of Siberia up to the Arctic Ocean in the north and the Bering Strait in the east, covering extensive peatland areas in the western and the north-east (Lena basin). We found a cumulative BA of 107.57 Mha between 2001 and 2023, with an average of 4.68 Mha/year and a large interannual variability (coefficient of variation = 57%). Fires were concentrated in central northern Siberia, over the eastern taiga ecosystem in the Republic of Sakha (Yakutia). This area is dominated by large extents of larch forests underlain by permafrost (Fig. 1, A and B and fig. S1). To a lesser extent, fires were also present in the subarctic southern Siberian taiga and the Arctic northeastern taiga as well as the easternmost tundra ecoregions. Three peak years of fire activity were observed, the most extreme years being during the recent period from 2019 to 2021. This period accounts for more than 25% of overall cumulative BA, with the average BA being 127% larger compared to the other years (Fig. 1C). The year 2021, during which fires burned 10.58 Mha, experienced the largest BA in our record, with substantial burning of larch forests underlain by permafrost. The 2019 to 2021 surge of extreme fire activity coincided with anomalous dry and hot summers. The occurrence of fire-promoting conditions was previously linked to an anomalous Arctic front jet (*10*). In general, more than 70% of fires occurred in early to mid-summer (June 21 to August 20) with a maximum in July. Spring fires usually ignite in May with small-scale burns after the expansion of snowmelt with BA being less than few tens of thousands of hectares. The exception to this pattern was the year 2011 that showed an anomalous uprising of spring fires despite a normal yearly BA (fig. S2A). In 2011, spring fires accounted for 59% of the total BA of that year (2.88 Mha). This anomaly can be attributed to an early snowmelt as the average April snow cover was substantially lower than in other years (fig. S2B), favoring lightning-related fire ignitions (*10*). The following year (2012) was an extreme summer fire year with the second-largest recorded annual BA. Most of Siberian BA resulted from large fires exceeding 1000 ha, which accounted for ~92% of the total BA in our study region (fig. S3A). In 2019 to 2021, the fraction of BA from large fires was even higher (94 to 96%). The temporal correlation between the number of large fires and annual BA was strong across all years, except in 2021 (fig. S3B), with an overall correlation of *r* = 0.74, which increases to 0.83 when excluding 2021. In that year, a megafire—the largest fire event in our record—accounted for 14% of the total BA, spreading over 1.5 Mha, approximately, from 5 June to 15 August 2021 (Fig. 2). This megafire coalesced from multiple ignitions, of which some were flares-ups of overwintering fires from 2020.

Overwintering fires that preceded this megafire and other large fires of 2021 persisted at the surface until late September 2020 and disappeared as surface fires during the 2020 to 2021 cold season yet survived in “zombie” mode (*29*, *30*) with slow belowground smoldering in carbon-rich organic soils (*29*) and reemerged to the surface in early June 2021 following snowmelt (Fig. 2). Peatland burning dominates those overwintering fires during 2020 to 2021 (Fig. 2). Overwintering was associated with the start of 48 of the 513 large fires recorded in 2021, including the megafire. Throughout our time series, several extremely large fire events were triggered using the same process (7.10% of large fires; see fig. S3A). Of 770 recorded overwintering fires, peatlands were overwhelmingly present within the buffer zone linking them with their presumed patches of origin (759 cases).

Overall, BA in our Landsat-based 30-m resolution dataset was found to be substantially larger than global coarse-resolution BA datasets derived from MODIS data, 42 and 66% larger than the MODIS-based FireCCI and MCD64A1 (collection 6), respectively (fig. S4A) (*15*, *16*, *31*). The overall accuracy was significantly higher in the Landsat-based dataset in comparison with both MODIS maps (see the full validation results in table S1). Our BA estimates were also significantly higher than the ones of GFED4s, which combines MODIS and active fire counts for the recent period (76% higher) (*32*) and the recent GFED500 dataset at 500 m (54% higher) (*14*), although these two GFED datasets both attempted to better account for small fires. Our long-term Landsat-based dataset also detected more Arctic BA than Descals *et al.* (*33*) who used Landsat and Sentinel-2 for the most recent years (fig. S4B).

### Arctic peat fires rising from recent Arctic warming

**Fig. 3.**
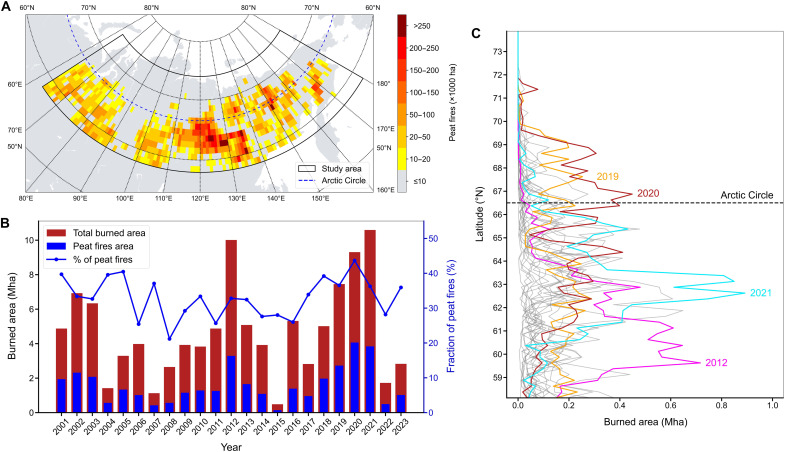
Contribution of Siberian peat fires during the period 2001 to 2023. (**A**) Total peat fires during the period 2001 to 2023 aggregated to 1° grid cells. (**B**) Yearly BA and the proportion of peat fires between 2001 and 2023. (**C**) Yearly frequentist distribution of peat fires by latitudes.

More than 25% of our study area (~240 Mha) was identified as peatlands (fig. S1B), and nearly one-third (32.96 ± 2.31%) of the BA occurred in peat-dominated regions. The classification of peat fires showed high agreement with visually interpreted images (see the Validation of burned area and peat fires mapping section in Materials and Methods). The total BA and the peatland BA were highly linked spatially and temporally, with the largest peat fires occurring in the east and northeast Siberian taiga as well as in the Russian Bering tundra (Fig. 3A). The interannual correlation between peatland and total BAs was 0.97. However, during the record-breaking heatwave and drought of 2020, peat fires represented an abnormally high fraction of total BA (43.6%; see Fig. 3B and fig. S5). This anomaly is explained by extensive peat fires in permafrost bogs within north-eastern taiga and the far north-eastern Siberia tundra. In 2020, peat fires affected 4 Mha of peatland bogs, which represents 1.6% of the total peatland area in our study domain, surpassing the impact of other extreme fire years over the entire Siberian domain in 2021 and 2012, when fires primarily occurred in regions south of the Arctic Circle that have a lower peatland and permafrost coverage (fig. S6). The fire season of 2020 was the most extreme within the Arctic Circle, with more than 26.85% of all recorded BA occurring in that year alone; the second record season being 2019 (Fig. 3C). In 2020, the Arctic experienced the highest number of large fires >1000 ha (207 of 1184 in total; fig. S7).

**Fig. 4.**
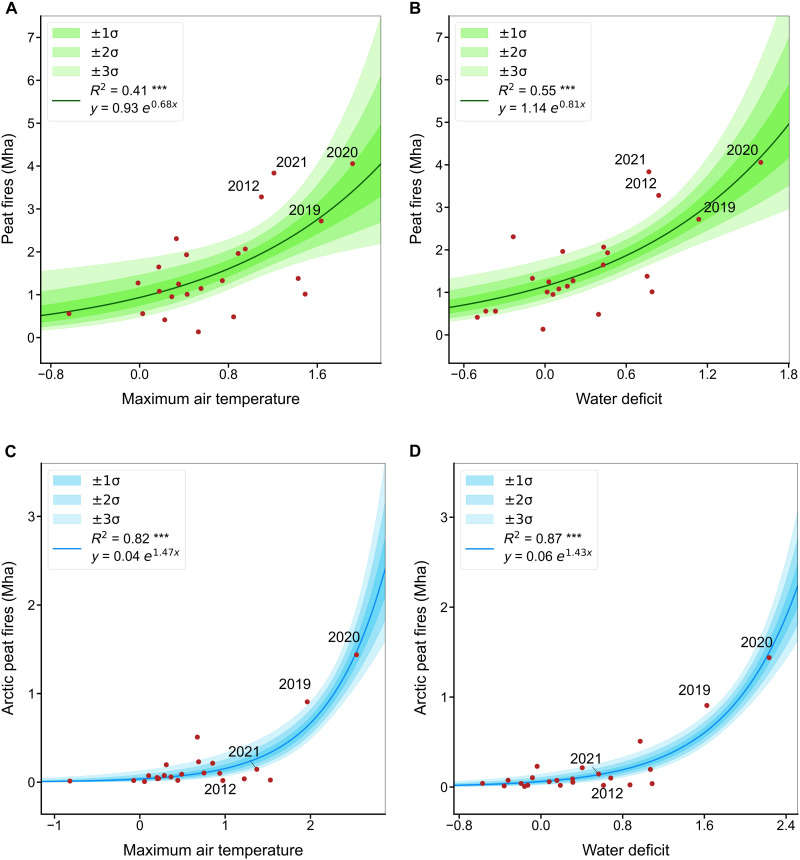
Regressions between main summer climate indicators (represented as anomalies from long-term means) and peat BA in Siberia between 2001 and 2023. Panels (**A**) and (**B**) represent relationships across the entire study area (>58° N), while panels (**C**) and (**D**) represent the relationships in Arctic Siberia. Comprehensive plots are in figs. S8 and S9. The long-term climate summer means were calculated for the 1958 to 2023 period. Solid regression curves are labeled by their corresponding coefficient of determination (***P < 0.001) and the equation of the generalized linear model. The shading indicates one, two, and three SDs from the regression line. The anomalies were computed using *z* scores.

Siberian peat fires exhibit an exponential increase in response to dry and warm summer climate conditions (Fig. 4, A and B). Exponential fits between peat-BAs and climate drivers were robust in a large set of climatic drivers (see fig. S8) and explain 41 to 58% of the interannual variability of peatland BA. Peat fires can be predicted accurately by indicators of soil moisture such as the climatic water deficit (defined as the difference between actual and potential evapotranspiration), soil moisture, and the Palmer Drought Severity Index (PDSI) and by fire weather variables known as the Drought Code (DC) and Duff Moisture Code (DMC). The DMC and DC are proxies of soil moisture in deep layers of soil (5 to 10 cm for DMC and 10 to 20 cm for DC) and the water table depth (*34*), which acts as the key buffer against fire propagation and persistent smoldering in peatlands (*35*, *36*). Notably, in the year 2020, marked by exceptionally high peat fire activity, all climatic variables have deviations larger than one SD from their long-term averages. This suggests that these extreme peat fires were mainly climate-driven. In the Arctic region, the relationship between climate anomalies and peatland BA was even stronger, with steeper exponential slopes and more consistent correlations across variables. For instance, the climatic water deficit and air temperature explained 0.87 and 0.82, respectively, of the variability of Arctic Siberian peat fires (Fig. 4, C and D, and see fig. S9 for all plots). This impact can be attributed to the high vulnerability of Arctic permafrost to the increase of temperatures compared to subarctic permafrost of boreal larch forests, which tend to exhibit greater resilience to disturbances and frequent fires.

### Burn depth and fire carbon emissions

As peat fires primarily consume soil organic matter, the most important variable to estimate emissions is the burn depth, which depends on fire intensity and peatland ecosystem characteristics. Burn depth was modeled using an eXtreme Gradient Boosting (XGBoost) (*37*) machine learning model trained on a field data collection of 894 sites (see Materials and Methods), which was then used in the predictions of belowground carbon combustion. Burn depth and carbon emissions are linearly linked but the slope of the linear relationship is influenced by site characteristics (fig. S10A). We also estimated aboveground carbon combustion using aboveground biomass maps (*38*) along with other predictors. Recursive feature elimination (RFE) was applied to choose the best predictors among more than 40 variables that encompass fire characteristics, climate, soil properties, topography, and vegetation cover. The predictions of burn depth performed the best with an *R*^2^ (coefficient of determination) of 0.5 (fig. S10B), while belowground carbon combustion had a smaller *R*^2^ of 0.26 (fig. S10C). Aboveground carbon combustion has an *R*^2^ of 0.45 (fig. S10D). All models showed higher performance for moderate values and low relative bias (*rBias*) from field observations.

**Fig. 5.**
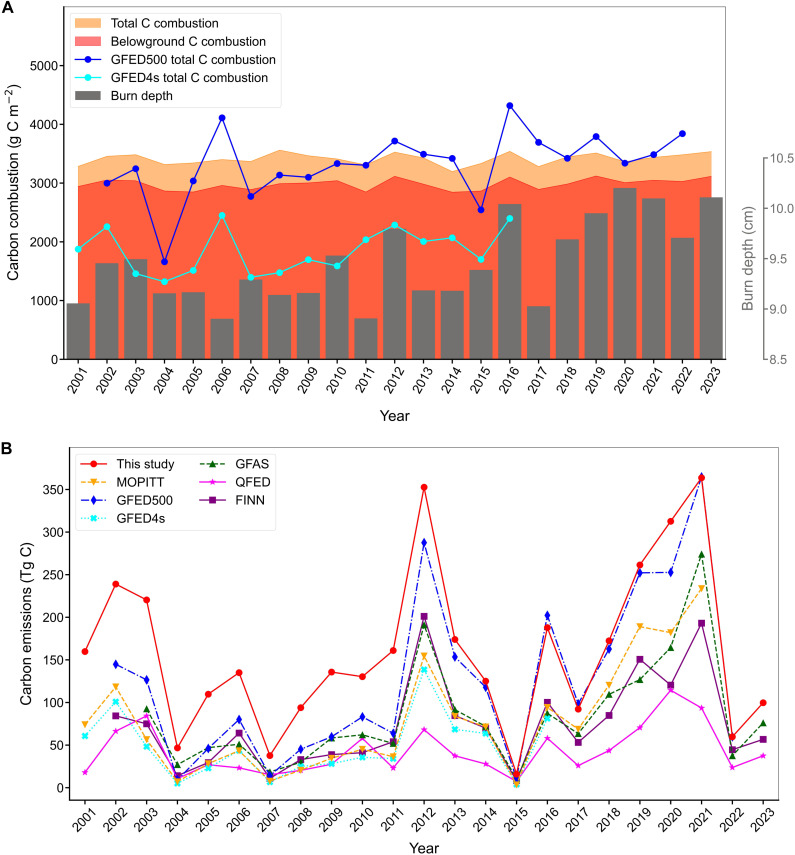
Comparison of trends in Siberian carbon fire emissions and burn depth between 2001 and 2023. (**A**) Trends in carbon combustion and burn depth compared to GFED4s and the new 500-m GFED carbon combustions. (**B**) Comparison of estimated annual Siberian fire emissions with common fire emission datasets.

In total, we estimated that all fires released a total of 3.69 ± 0.15 Pg C between 2001 and 2023, with peat fires contributing to approximately one-third (1.24 ± 0.06 Pg C). Across all fire types (peat and nonpeat), the annual carbon emissions per BA averaged 3427 ± 140 g C m^−2^ (means ± SD) (Fig. 5A), in agreement with recent estimates of carbon combustion in Eastern Siberian larch-dominated forests. For instance, Veraverbeke *et al.* (*12*) reported emissions rates of 3360 ± 930 g C m^−2^, primarily from belowground sources. Based on these field measurements, Delcourt *et al.* (*39*) estimated an average carbon combustion of 3200 ± 750 g C m^−2^ over two large fires in Siberia, with ~78% (2490 ± 560 g C m^−2^) originating from belowground pools. Our findings show that 88% of the emissions (3010 ± 139 g C m^−2^) were released from belowground combustion, slightly larger than the previous study but still within their reported confidence range. The annual burn depth ranged between 8.90 and 10.20 cm. While the interannual variation of carbon combustion was low, burn depth showed a small but significant increasing trend reaching up to 0.04 cm year^−1^ (Mann-Kendall test’s *P* = 0.007). In comparison with GFED datasets, our carbon combustion estimates were slightly higher than those of GFED500 (3321 ± 553 g C m^−2^; Fig. 5A) (*14*) that exhibited an increasing trend over time (Mann-Kendall test’s *P* = 0.006). In contrast, GFED4s estimates were significantly lower, with our results nearly double (92% larger) than those of GFED4s. This discrepancy is likely due to the limited field samples in boreal and Arctic Asia used in GFED4s to estimate belowground combustion (*12*, *14*). In terms of total carbon emissions, our estimates were marginally higher than GFED500 in most recent years (Fig. 5B), while the corresponding BA estimates were relatively similar (fig. S3). For earlier years, our BA detections were significantly larger than GFED500, leading to higher emissions. Over the entire period covered by both datasets (2002 to 2022), we report 30% more carbon emissions than GFED500. Compared to GFED4s, our emissions were more than three times higher for the period 2001 to 2016. Similarly, our emissions were substantially higher than those of other global fire emission datasets (Fig. 5B). For peat fire carbon emissions, our estimates are substantially higher than those reported by GFED4s, with pronounced peaks in 2012 and fire seasons between 2019 and 2021, in agreement with the peat fires extent. In contrast, peat fires account for merely 2.39% of total fire emissions reported in GFED4s against one-third in this study (fig. S11B). This suggests that the contributions from peatland to total fire emissions are coarsely underestimated in GFED datasets, most likely due to the omission of peatlands in their land cover classification (*40*). This bias is also present in the updated GFED5 product (“Beta” version), which has more small fires but attributes only 0.46 to 5.50% of them to peatland (0.86% in 2020), far below the 21.16 to 43.65% peat fire fractions reported in our study, with the peak occurring in 2020 (fig. S11A).

### Causal links between climate and peat fires

**Fig. 6.**
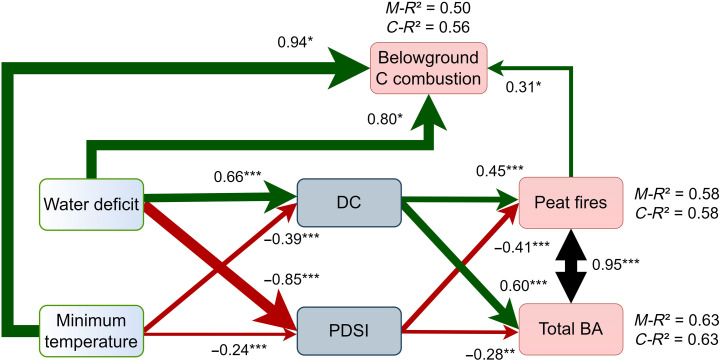
Causality network of climatic factors driving Siberian fires. The width of connecting lines indicates the magnitude of the effect derived from SEM model, and the double arrow line represents covariance. *M-R*^2^ is the marginal *R*^2^ of each LMM, representing the variability of the endogen variable explained by fixed effects alone. *C-R*^2^ is the conditional *R*^2^ that indicates the variance explained using both fixed and random effects. Green lines denote positive effects, and red lines denote negative effects. (***P < 0.001, **P < 0.005, and *P < 0.05). DC, Drought Code; PDSI, Palmer Drought Severity Index.

We used piecewise structural equation model (SEM) to examine the significance of causal relationships among key climate and fire weather variables during the summer season and their influence on the interannual variability of peat fires and belowground carbon combustion (Fig. 6). Linear mixed-effects models (LMMs) were used to assess the marginal and conditional explanatory power of each model across three subregions. The model goodness was evaluated using Fischer’s *C* test (*C*_16_ = 20.49, *P* = 0.2). This analysis shows that water deficit is a major climatic driver of peat fires and emissions, whereas adding minimum temperature improves the level of variance explained by the model, and was particularly linked with belowground carbon emissions from peat fires. The additional impact of temperature is aligned with a recent study (*9*) showing the existence of a minimum threshold of environmental temperatures that allow a self-sustained smoldering fire on the peatland soils. The LLMs explained 63 and 58% of the overall variability of total BA and peat fires with both marginal and conditional *R^2^* equal, suggesting that climate is the main driver controlling fires over the entire Siberian region with less impact of random effects (i.e., the vegetation zones in our case). These endogenous variables were highly correlated (*cov* = 0.95); however, the drivers slightly differed. The DC dominated the variation of total BA (standardized regression coefficient β = 0.60), while DC and long-term drought, represented here by the PDSI, contributed almost equally in the case of peat fires. The model also reveals that an increase in peat fires in combination with dry conditions (water deficit) might increase the chances for larger belowground carbon combustion. More than 50% of the variability of belowground carbon combustion was explained in this path even with fixed effects alone and increased to 56% by including random effects. The link between fire weather and water deficit was strong as the latter indicator was highly linked to DC (β = 0.66) and PDSI. The link with the latter variable was negative (β = −0.85) as negative values of PDSI translate to dry conditions. We used simple linear regression for these paths and the models explained 69 and 71% of the variations of DC and PDSI, respectively. The minimum temperature was negatively linked with the increase of DC due to the high values of minimum temperature in western Siberia that were not correlated with fire weather conditions (fig. S12).

## DISCUSSION

Carbon-rich ecosystems of Siberia could become a future hot spot of fire-induced carbon losses driven by the increasing frequency of extreme dry seasons, which translates into extreme fire years (*33*) and offsets CO_2_ ecosystem uptakes (*41*). The surge in Siberian fires in the 2019 to 2021 period was a clear demonstration of the impacts of extreme dry weather conditions. Our higher-resolution BA dataset detected more BA than coarse-resolution datasets, typically biased toward the underestimation of BA extent (*31*). The BA maps presented here are produced using a hybrid approach that combines active fire data with Landsat imagery, improving the accuracy and spatial detail of BA mapping. We also mapped more BAs than those detected in a recent map of Siberian Arctic fires derived from Landsat and Sentinel-2 high-resolution sensors complemented by coarse-resolution BA products for the pre-2013 period (*33*). These omissions could be attributed to the lower observation frequency of high-resolution satellites, which has major implications on fire detection in regions with persistent cloud cover such as Siberia (*42*, *43*).

Extreme peat fire years, such as 2020, not only resulted in substantial carbon emissions but also promoted the persistence of peat fires into the following season through overwintering (*30*, *44*). In our analysis, we found that the largest fire event in the dataset (in 2021) was driven by multiple reignitions occurring adjacent to the previous season’s fire patches that likely sustained by slow, belowground smoldering over winter. A similar pattern of winter holdover reignitions was also observed in other exceptionally large fire events.

Global fire emission datasets, such as GFED products, appear to underestimate peat fires, which leads to a large underestimation of the impact of these fires on peatland carbon pools. In recent years, our estimates of carbon emissions from Siberian wildfires generally agree better with those derived from the 500-m GFED model. However, during earlier years, the lower estimations of BA in GFED500 led to an underestimation of fire-related carbon emissions as compared to this dataset. Following the conclusions of recent studies (*14*, *16*, *23*), our carbon combustion modeling framework focused on achieving the highest possible spatial resolution (up to 30 m in the Arctic-boreal region) to capture the small-scale combustion heterogeneity caused by fire severity, vegetation cover, and soil properties within single fire patches, which could complement the coarser-resolution climate variability (1/24°; ~4 km). This high-resolution modeling approach allowed us to improve estimates of Siberian wildfire emissions and to identify the fraction of peat fire emissions with a high accuracy compared to standard fire emission models. While the additional BA detections largely explain the higher overall fire emission estimates relative to other datasets, our peatland classification provides a more refined picture of the direct impacts of peat fires and offers a foundation to further assess their cascading effects on permafrost. In addition to releasing substantial amounts of carbon, peat fires initiate long-lasting ecological disturbances, which eventually amplify future losses of peatland carbon sinks and impact climate feedback. While this process was not accounted for in this study, peat fires accelerate permafrost thaw through the expansion of thermokarst bogs, particularly along peatland edges, where permafrost could become irrecoverable even decades after burning (*11*). The anaerobic conditions associated with thermokarst bog formation potentially increase the rate of methane emissions (*45*). Moreover, peat fires consume the insulating moss and organic surface layers, resulting in warmer soils and a deeper active layer (*46*–*48*), which increases the rates of soil carbon dioxide respiration (*11*). The combustion of surface biomass also alters energy balances by reducing evapotranspiration and short-term summertime albedo (*49*), which increases ground heat flux and could cause an additional expansion of active layer, with direct climate feedbacks (*11*, *50*).

Our SEM causality model confirms the role of climate as the main driver controlling the interannual variability of Siberian fires and their subsequent damage. Our findings suggest that the magnitude of peat fires accelerates as a response to climate anomalies, particularly above the Arctic Circle, where the links were stronger, and the sensitivity of peat fires to climate was higher than in the subarctic. This highlights the greater vulnerability of Arctic ecosystems to climate warming compared to the subarctic regions, which exhibit higher resilience due to the symbiotic relationships associated with the taiga-permafrost coupled system (*51*). This system tends to inhibit fires during dry periods by intercepting sunlight, maintaining soil moisture, and preserving ground ice (*51*, *52*). Consequently, the rise in extreme climate anomalies significantly amplifies the likelihood of catastrophic Arctic fires, exposing substantial amounts of long-term organic carbon sinks to decomposition through permafrost thaw and warmer conditions (*11*), and abruptly increasing the risk of more frequent and severe wildfires in the future (*47*, *53*).

The findings of this work reveal that peat fires represent a major and substantially underestimated source of carbon emissions in Arctic-boreal regions, with an extreme sensitivity to climatic anomalies. This highlights the critical vulnerability of northern ecosystems, particularly carbon-rich peatlands, and raises more concerns about their uncertain resilience under projected future warming. Our results provide valuable benchmark for improving the representation of peatland processes in Earth System Models, thereby enhancing simulations and monitoring of peatland carbon balance in northern ecosystems.

## MATERIALS AND METHODS

This study integrates remote sensing, spatial modeling, and statistical analyses to assess Siberian fire activity, especially in peatlands, and their associated carbon emissions. The methodology section is organized into six main components: (i) the generation of BA maps, including all post-processing and enhancement steps; (ii) the creation of high-resolution peatland maps and the resulting classification of peat versus nonpeat fires; (iii) the validation of both BA and peat fire maps; (iv) the modeling of burning depth and carbon combustion, including model evaluation and uncertainty assessment; (v) the description of different fire emissions datasets involved in the comparison with our carbon emission estimates; and last (vi) the exploration of the causal relationships between climate variables and peat fires.

### Burned area generation

#### Preliminary BA classification

The process of generating BA maps was developed using the Burned Area Mapping Tools (BAMT) cartography tool, version 1.7 (*54*) as a starting point. This algorithm is a semi-automatic procedure in the Google Earth Engine (GEE) platform that allows to map fires using Landsat and/or Sentinel-2 multispectral datasets in an interactive way that involves recursive addition of training samples (burned and unburned) that are trained over image composites using a random forest (RF) classifier while visually monitoring the performance of the resulting BA map until the results of the classification are optimal. The original algorithm is adequate for small-scale mapping but less practical for large spatiotemporal extents. In our case, the study area spans from 63°E, 58°N (longitude, latitude) to 180°E, 74°N (areas north of 74°N rarely burn and were excluded from the study), covering around 9 million km^2^ and extends for 23 years (2001 to 2023), which makes this version of BAMT tool less useful due to recursive training repeated for several years and subsets (GEE would fail to process and visualize such a large extent at once). With the exception of the training sample collection, best handled within the GEE environment, the updated algorithm was entirely developed in Python for better flexibility and automation. Moreover, the processing performance was significantly improved through the implementation of some parts of the postprocessing workflow locally outside GEE.

We assumed that fire signal changes spatially based on land cover type and collected extensive training samples for the year 2019 and used this year as a reference to train a RF classifier (nTrees: 200, MinLeafPopulation: 10, MaxNodes: 450, VariablesPerSplit: “sqrt”, BagFraction: 0.5), and then this model was used to classify all years assuming the independence between fire signal and time dimension.

The algorithm of BA mapping used the entire available imagery of Landsat sensors (Landsat-4, -5, -7, -8, and -9). The data were processed at a 30-m spatial resolution, covering the visible, near-infrared (NIR), and shortwave infrared (SWIR) spectral ranges. Among the available Landsat products in GEE, the collection 2 (C2) Landsat tier 1 surface reflectance (SR) product was selected. This product provides atmospherically corrected and orthorectified surface reflectance data. We used bands of the visible (blue, green and red) and NIR regions and two bands in the SWIR (short and long SWIR), in addition to three spectral indices, defined as

$$\text{NDVI}=\frac{\rho_{\text{NIR}}-\rho_{\text{Red}}}{\rho_{\text{NIR}}+\rho_{\text{Red}}}$$

$$\text{NBR}=\frac{\rho_{\text{NIR}}-\rho_{\text{SWIRL}}}{\rho_{\text{NIR}}+\rho_{\text{SWIRL}}}$$

$$\text{NBR}2=\frac{\rho_{\text{SWIRS}}-\rho_{\text{SWIRL}}}{\rho_{\text{SWIRS}}+\rho_{\text{SWIRL}}}$$

where NDVI denotes Normalized Difference Vegetation Index, NBR denotes Normalized Burned Ratio, NBR2 denotes Normalized Burned Ratio 2, ρ_Red_ denotes reflectance in the red band, ρ_NIR_ denotes the reflectance in the NIR band, ρ_SWIRS_ denotes reflectance in the short SWIR band, and ρ_SWIRL_ denotes reflectance in the long SWIR band.

After computing spectral indices for each scene, the minimum NBR was used to create yearly composites (pre- and postcomposites) as it represents the highest spectral fire signal (*54*). Then, for each year denoted *t*, the difference image composite is computed between post- and precomposites (composite*_t_ −* composite_*t*−1_). Although not crucial in our case since the yearly image composites extend from March to the end of November, we added a date difference band defined as the difference of time between compositing dates for each pixel. This band is important in case the image composites are successive to mitigate confusions in double burns. Large date differences might allow double burns in some ecosystems, but not during short periods of less than 3 months. The time band is not important in the Siberian case; however, it was added to make the algorithm adaptable for different terrestrial ecosystems. In the end, a total of 28 features were involved in the classification.

We established a preliminary sampling design that specifies a minimum number of samples (small quadrilateral polygons of approximately 5 to 20 pixels width) fixed at 120 samples of each class, stratified over the different land cover types (*55*), proportionally to the corresponding total BA of each land cover derived from FireCCIS311 BA product of the year 2019 (*56*, *57*). These 240 samples were distributed onto the processing system (3° by 2° tiles) with the maximum spread possible to cover distinct areas. More samples were recursively added to distinct tiles, representing different scar signals (new tiles were added to the first selection) until the classification results were stabilized and optimal visual accuracy was obtained. In the end, a total of 761 samples (402 burned and 359 unburned) were collected.

The atmospheric correction of Landsat SR based on the FMask algorithm (*58*) generally provides consistent cloud masking. However, in some cases, this mask fails and might lead to substantial confusion for BA mapping due to the saturation of NIR and SWIRL bands in pixels contaminated by clouds. Therefore, we applied an additional restrictive filter to mitigate this discrepancy using a maximal threshold of 0.3 for SWIRL and 0.33 for NIR bands. Another filter was applied for images of Landsat-5 collected in 2001 and 2002 to mitigate the impact of diagonal strips of abnormally high SWIRL, and low SWIRS observed mainly along the edges of the scene footprint, leading to incorrect fire detections with high signal in NBR2 index. The anomaly perhaps resulted from the internal synchronization malfunction that arose from long-term wear of the primary scan mirror mechanism of Landsat-5, later changed to a backup mode in 2002 (*59*). This issue was resolved by applying a restrictive minimum of −0.3 for NBR2. Last, pixels with red or NIR values lower than 0.005 are discarded as they are mainly covered by water and potentially lead to commission errors.

To reduce omission, particularly inside fire patches, and commission errors caused by uncertain patches and isolated pixels (salt and pepper noise), several postprocessing procedures were applied. First, a growing region was implemented in GEE using unrestrictive rules applied to the resulting probability of burn in addition to some predictors, as follows

$$\left\{P_{b}\geq 40\%\right\}$$

$$\text{OR}\;\{\text{NBR}2_{\text{post}}\;\leq 0.06\;\text{AND}\;\text{NBR}2_{\text{diff}}\;\leq 0.005\;\text{AND}$$

$${\text{Red}}_{\text{diff}}\geq 0.04\;\text{AND}\;{\text{NIR}}_{\text{diff}}\leq -0.003\}$$

$$\text{OR}\;\left\{\text{NBR}2_{\text{post}}\leq 0\;\text{AND}\;\text{NBR}2_{\text{diff}}\;\leq 0.01\;\text{AND}\;{\text{Red}}_{\text{post}}\leq 0.03\right\}\mathrm{ccc}$$

where *P*_b_ denotes the probability of burn derived from the RF classifier and the subscripts post and diff (difference) represent the source image composite of the band feature. This growing region was applied exclusively within the vicinity (2 km) of confirmed burned seeds, whose *P*_b_ is higher than the average probability of the burned training class. In the second step aiming to mitigate commission errors, burned patches with less than four seeds were discarded. Then, successive morphological opening (dilation followed by erosion) and closing (erosion followed by dilation) were applied, in this respective order, using 90 mm by 90 m kernels to ensure consistent patchiness. The last two steps were processed locally after downloading GEE results.

#### Burned area enhancement using active fires

Fire signals can last long periods in boreal ecosystems; however, the abundant cloud coverage throughout the year makes the satellite revisit time a crucial factor. The MODIS and VIIRS sensors collect at least 16 times more daytime observations than a single Landsat sensor, which allows notably higher potential to detect fires if they extend over large swaths. This difference was pronounced in 2012, which was the year with the second highest BA, coinciding with the decommissioning of Landsat-5, leaving Landsat-7 alone with all its corresponding issues (mainly the stripping effects caused by Scan Line Corrector failure observed since May 2003). On the other hand, one of the objectives of this dataset is to derive fire patches with consistent dating, which are generally heterogeneous due to the late detection of some fire patch parts obscured for long periods by clouds. To overcome these two issues, we used active fires derived from the MODIS MCD14ML product at ~1000-m spatial resolution (before 2012) and the VIIRS VNP14IMGML product at ~375-m spatial resolution (2012 onward) to reassign the most appropriate burning date using neighbor active fires (Voronoi polygons). For the year 2022, gaps were observed in data collected from VIIRS sensor onboard the joint NASA/NOAA Suomi-National Polar orbiting Partnership (S-NPP) satellite. Therefore, the VJ114IMG product, derived from a similar VIIRS sensor onboard NOAA-20 (JPSS-1) satellite, was used as a source of active fires. We rasterised these active fires based on the corresponding along-track and cross-track resolutions of each active fire point. Each pixel connected to a preliminary burned patch (Queen’s case contiguity) was amended to the patch if it was not dominated by water throughout the year (fig. S13). A threshold of annual water cover percent of 80% was used to eliminate these pixels based on the Global Land Analysis and Discovery water dataset (*60*). Sometimes, BA detections, especially of late fires, were detected the next year due to the lack of postfire observations. To avoid these cases, a burned pixel was relocated to the previous year if it satisfied each of the following conditions:

1) There is at least one active fire in 5 km proximity in the previous year.

2) The distance to the nearest previous year’s active fire is lower than the distance to active fires of the processing year.

#### Generation of fire patches

Burned pixels can be clustered using their spatial and temporal relationships into unified fire patches. This clustering enables the assessment of individual fires in terms of size, duration, propagation speed, etc. (*61*–*63*). These clustering approaches are generally based on the “flood-fill” algorithm (*64*), which aggregates contiguous pixels based on a cut-off value, defined in our case as the maximum duration between two neighboring pixels to be considered as part of the same fire event. In Arctic-boreal ecosystems, it is recommended to use large cut-off values as they represent infrequently burning landscapes with slower fire spread rates, fewer ignitions, and greater obscuration by clouds (*63*). After multiple trials (12 to 24 days), a cut-off value of 16 days was considered as optimal. To preserve the integrity of large fire events spanning multiple processing units (3° by 2° tile), each tile was processed within a rolling window of nine tiles centered on the target tile and then duplicated patches were removed. To ensure capturing patches separated by rivers or other topographic barriers, a buffer of ~150 m was applied. The resulting fire patches include information about size, duration, start and end day, and the fraction of peat fires.

In this study, we also analyzed the proportion of large fires that flared up as a result of overwintering process. These wildfires are presumed to extinguish on the surface at the end of the fire season but survive through the winter by smoldering belowground, sustained by carbon-rich soils, before reemerging at the surface when favorable weather conditions return (*29*, *30*, *44*). We assumed that large fires caused by overwintering reignite at the surface before the start of the main fire season (July 1) and should reappear in a maximum vicinity of 1000 m from previous season fires (*30*).

### Peatland map downscaling and peat fire classification

We developed a peatland classification at 90-m resolution (F.A. and T.H.N., in preparation) using as inputs Landsat surface reflectances, topography-related information, and water table data (*65*). The classification is based on a neural network (NN) classification trained on the PEATMAP dataset (*25*). Once trained, the NN provides a continuous peatland index between 0 and 1, giving a proxy of peatland presence plausibility. This peatland index was then used to downscale the PEATMAP dataset (1 km) at a higher spatial resolution so that the pixels with the highest high-resolution peatland index sum up the same area as the PEATMAP coarse-resolution one. This downscaling process has been developed for all boreal regions where PEATMAP is at much lower resolution, over large and irregular polynomial shapes.

This definition of peatlands in the PEATMAP dataset encompasses various wetland formations, including, mires, forested bog, nonforested bog, forested swamp, nonforested swamp, alluvial formation, etc. [see (*25*) for a detailed description of the field data classes used and their sources]. The source PEATMAP dataset shows a strong consistency across northern latitudes with statistics of the International Mire Conservation Group Global Peatland Database (*66*), which is based on national inventories. The resulting peatland map was resampled (nearest neighbor) to a spatial resolution of 0.00025° (~30 m) to ensure alignment with the BA dataset, allowing us to classify fires as occurring in peat or nonpeat areas.

### Validation of BA and peat fires mapping

To ensure accuracy in peat fire classification, we manually interpreted and labeled fires that consumed peatlands using the Sentinel-2 images for the years 2017 to 2022. Peatlands predominantly undergo smoldering combustion during wildfires, characterized by slow, flameless burning (*35*, *67*). Therefore, the identification of peat fires relied primarily on detecting smoke plumes and interpreting landscapes in Sentinel-2 images. To classify a fire as a peat fire, it must meet the following criteria

1) Smoke must persist for over a week.

2) The area was not densely forested before the fire.

3) The terrain is typically flat, which implies that it could be wet or it is located near standing water.

This procedure allowed to identify a collection of 4274 peat fire locations, of which more than 99% (4237) were in perfect agreement with our map of peat fires. The overall mapping of BA was validated against the Burned Area Reference Database (*68*, *69*). Over the period 2003 to 2023 (note that reference units for 2020 to 2023 are pending publication), this dataset included 35 reference units with fire events falling in our study domain. The validation exercise revealed omission errors of 10.41% and commission errors of 29.46%, with a dice coefficient of 0.79. Coarse-resolution BA products showed significantly lower overall accuracy with dice coefficients of 0.71 and 0.73 for MCD64A1 (collection 6) (*70*) and FireCCI51 (*71*), respectively. See table S1 for a full comparison of performances of the three datasets.

### Modeling of burning depth and carbon combustion

Standard bottom-up approaches of fire emissions modeling such as GFED [e.g., GFED4s (*32*)] use the Seiler and Crutzen (*72*) equation of carbon fluxes from biomass burning, defined as

$$E_{i}=\mathrm{BA}*\mathrm{FL}*\text{CC}*{\mathrm{EF}}_{i}$$

where *E_i_* and EF*_i_* denote the estimated emissions and emission factor corresponding to the element *i*, respectively. CC is the combustion completeness and FL is the fuel load. The latter parameter could be distributed to aboveground pools (woody debris, stems, fine litter, etc.) simulated using vegetation parameters and belowground pools (e.g., SOC) that are mainly simulated using burn depth and soil bulk density. Because of the difficulties related to deriving accurate dynamic FL, CC, and EF, simplified assumptions are established to estimate these values, which might generate large uncertainties. In this work, we used 894 field measurements derived from literature (*73*, *74*) that cover different northern fire regions, including Alaska (*n* = 285), Canada (*n* = 568), and Siberia (*n* = 41). These measurements were used to train XGBoost (*37*) machine learning models, incorporating more than 40 variables related to fire severity characteristics, climate, soil properties, topography, and vegetation cover (table S2). Coarse-resolution predictors were resampled (nearest neighbor) to the resolution of BA (0.00025°). Three models were developed; the first one aims to predict burn depth, while the other two correspond to aboveground and belowground carbon combustion. Here, we define carbon combustion as carbon emissions from fires per unit of BA (i.e., in g C m^−2^). The two carbon combustion types (belowground and aboveground) were separated as they were influenced by distinct yet overlapping sets of parameters. Burn depth is widely used as a proxy of belowground carbon combustion (*75*, *76*) and demonstrated a linear relationship with belowground carbon combustion based on our field data (*r* = 0.75). A small part of belowground carbon combustion variability depends on field data provenance (fig. S10A). Therefore, burn depth was used in the model of belowground carbon combustion. In the case of aboveground combustion, aboveground biomass information (*38*) of the year 2010, considered the closest available year to the time-series midpoint, was added. A comprehensive list of predictors used in the models is provided in table S2 along with their data source. Model selection was conducted using a cross-validation approach combining feature selection and hyperparameter optimization. Hyperparameters were tuned through Hyperopt Python library (*77*), a Bayesian optimization framework that was used to recursively sample 200 combinations of eight critical XGBoost hyperparameters. Hyperopt efficiently explores the hyperparameters’ probabilistic spaces by modeling a minimization objective function based on tree-structured Parzen estimator algorithm (*78*). Feature selection was implemented using RFE with each subset of features evaluated via 10-fold cross-validation, the average root mean square error was used as the optimization metric for Hyperopt. Model evaluation was carried out using 10-fold predictions repeated 100 times, generating 100 independent predictions for each site. These predictions were averaged and evaluated against observed values. Of the 42 predictors, 25 were retained to predict burn depth. The averaged values generated using this model in the evaluation phase were used in combination with eight additional features selected to predict belowground carbon combustion. For aboveground carbon combustion, 20 of the 43 predictors were selected.

The uncertainty in carbon emissions estimates was derived from the distribution of residuals of the carbon combustion models (i.e., the difference between predictions and ground truth computed in model evaluation phase) and propagated using a Gaussian process (GP). We adopted the Matérn kernel (*79*), a widely used approach for estimating covariance in spatial statistics (*79*–*81*). Unlike standard radial basis function kernels, the Matérn kernel allows for greater flexibility in capturing local smoothness of spatial data (*80*), which makes it more convenient for geospatial GP modeling. This kernel is defined as follows

$$k_{\text{Matern}}\left(x,x^{\prime }\right)=\frac{2^{1-\nu }}{\Gamma \left(\nu \right)}{\left(\sqrt{2\nu }\frac{∥x-x^{\prime }∥}{\ell }\right)}^{\nu }K_{\nu }\left(\sqrt{2\nu }\frac{∥x-x^{\prime }∥}{\ell }\right)$$

with the length scale ℓ representing the distance of decay and ν representing the smoothness of the kernel. Γ(ν) is the gamma function and $K_{\nu }$ denotes the modified Bessel function of the first kind of order ν. In this study, a value of 3/2 was selected for ν as it showed good performance for modeling physical processes (*82*), ensuring an adequate balance between smoothness and local variability. The Matérn kernel used to calculate priors can be defined as

$$k_{3/2}\left(x,x^{\prime }\right)=\left(1+\frac{\sqrt{3}∥x-x^{\prime }∥}{\ell }\right)\;\text{exp}\left(-\frac{\sqrt{3}∥x-x^{\prime }∥}{\ell }\right)$$

We assume that the residuals of carbon combustion estimates follow a normal distribution $\varepsilon =N\left(\mu_{\text{res}},\sigma_{\text{CC},\text{res}}^{2}\right)$, where $\mu_{\text{res}}$ and $\sigma_{\text{CC},\text{res}}^{2}$ are the mean and variance of residuals derived from model evaluations. For each pixel i with an area $A_{i}$ and a carbon combustion variance $\sigma_{\text{CC},i}^{2}$ drawn from a set of yearly burned pixels $X=x_{1},x_{2},\ldots ,x_{n}$, the scaled variance of carbon emissions can be expressed as $\sigma_{i}^{2}={\left(\sigma_{\text{CC},i}*A_{i}\right)}^{2}$. The covariance prior of X, including the heteroscedastic variance noise $D=\text{diag}\left(\sigma_{1}^{2},\sigma_{2}^{2},\ldots ,\sigma_{n}^{2}\right)$, is calculated as

$$k_{\text{prior}}\left(X,X\right)=k\left(X,X\right)+D$$

The posterior covariance can be calculated then as

$$k_{\text{posterior}}\left(X,X\right)=k\left(X,X\right)-k\left(X,X\right)\;k_{\text{prior}}{\left(X,X\right)}^{-1}\;k\left(X,X\right)$$

The computation of total covariance using GP has a time complexity of $O\left(n^{3}\right)$, which is not convenient for very large datasets (i.e., millions of burned pixels in our case). We used the Nyström approximation (*83*) to construct a low-rank approximation of the eigenvalues/vectors of the kernel matrix K (*n* × *n*) based on a low-rank matrix of m representative inducing points (m≪n). First, let the matrix K be decomposed as

$$K=\left[\begin{array}{cc} K_{\mathrm{mm}} & K_{m\left(n-m\right)}\\ K_{\left(n-m\right)m} & K_{\left(n-m\right)\left(n-m\right)} \end{array}\right]$$

where the upper *m* × *n* block is denoted $K_{\mathrm{mn}}$ and its transpose is referred to as $K_{\mathrm{nm}}$ that contains the cross-kernel evaluations between all *n* points of the matrix and the *m* inducing points. The matrix Kis a Gram matrix (*83*), which can be then approximated with a computation complexity of $O\left(m^{2}n\right)$ as

$$\tilde{K}=K_{\mathrm{nm}}\;K_{\mathrm{mm}}^{-1}\;K_{\mathrm{mn}}$$

Following this method, we approximate the kernel k(X,X) using the inducing set of burned pixels $Z=\left\{x_{1},x_{2},\ldots ,x_{m}\right\}$ as

$$k\left(X,X\right)\approx k\left(X,Z\right)\;k{\left(Z,Z\right)}^{-1}\;k\left(Z,X\right)$$

The accuracy of the Nyström approximation depends on the number *m* of inducing points and their ability to represent the spatial pattern of the data. Here, in this study, we selected 1000 randomly sampled inducing points per computation batch. Each batch corresponds to the set of burned pixels within a processing tile. This tile-specific sampling ensures adequate representativeness of spatial patterns while maintaining computational efficiency and allows to avoid using a large set of global inducing points distributed across the entire study area. The length of decay ℓ was set to 25 km (i.e., the maximum resolution of predictor variables), beyond which spatial correlations between pixels were ignored.

### Comparison of carbon emissions with common fire emission datasets

Several operational fire emissions datasets have been developed in recent years. Yet, they rely extensively on the MODIS coarse-resolution satellite data. For instance, the GFED (*14*, *32*) follows a bottom-up modeling approach based on MODIS BA (MCD64A1) (*84*). To account for small fires, distinct calibration strategies were applied depending on the version (*14*, *15*, *21*). Fire emissions are then estimated by combining the calibrated BA with fuel loads (leaves, stems, coarse woody debris, fine litter, and SOC) modeled using the Carnegie-Ames-Stanford Approach biosphere model (*85*, *86*) and constrained by combustion completeness rates. Other emission models build upon the direct relationship observed between fire radiative power derived from MODIS active fires and fire emissions (*87*). However, these methods are less appropriate for evaluating belowground smoldering fires at low ground temperatures that is a characteristic of peat fires, leading to significant underestimations (*88*). This family of datasets includes the Global Fire Assimilation System [GFAS; (*89*)] and the Quick Fire Emissions Dataset [QFED; (*90*)]. The FIre INventory (FINN) from the National Center for Atmospheric Research (NCAR) (*91*, *92*) uses MODIS active fires in addition to some other ancillary data to estimate BA, while the fire emissions modeling framework relies on chemical transport models. Recent works have derived fire carbon emissions through inversions of atmospheric carbon monoxide (CO) concentrations retrieved from the Measurements of Pollution in the Troposphere (MOPITT) in combination with dynamic combustion ratios of CO_2_ to CO (*17*, *93*). This dataset provides CO and CO_2_ emissions for the period 2001 to 2021. We used a static methane (CH_4_) to CO_2_ combustion ratio of 0.01 to complement it based on the findings of Wiggins *et al.* (*94*). The GFED4s dataset (*32*) covered the period 2001 to 2016 in our analysis, while the new 500-m model of GFED [GFED500; (*14*)] extends for the period 2002 to 2022. GFED5 “beta” version (*21*) was only used to compare peat fires fractions (2002 to 2020) as this information is not available in other GFED datasets. Emissions estimates in this new GFED dataset remain preliminary. GFAS (version 1.2; from 2003 onward) and QFED (version 2.6r; from 2000 onward) are near real-time products and the FINN dataset covers the period 2002 to 2023. All these datasets were included in our comparisons.

### Exploration of causal relationships between climate and peat fires

Studies confirmed that climate anomalies are prominently linked to Arctic-boreal wildfires (*10*, *33*) and to some degree linked to carbon combustion (*95*). Here, we used piecewise SEMs of the “piecewiseSEM” R package (*96*) to investigate the causal relationships between climate variables, fire weather, fires (total BA and peatland BA), and belowground peat fire carbon combustion. In comparison with standard SEM, piecewise SEM allows a variety of models such as generalized linear models and LMMs in addition to linear models. We focused on the LMMs to account for the random effects associated with distinct geographical Siberian zones along with fixed effects. Western Siberia lowlands (63°E to 90°E) are dominated by carbon-rich peatlands with a temperate climate strongly influenced by North Atlantic Oscillation (*97*). Central Siberia (90°E to 130°E), spanning the Central Siberian Plateau to the western river valleys of the Sakha Republic, is characterized by larch forests with a presence of permafrost and experiences a harsh continental climate (*98*). The eastern zone (130°E to 180°E) is predominantly covered by eastern Siberian taiga transitioning into tundra toward the far northeast. This zone is marked by mountain ranges with an extremely continental climate of long and cold winters followed by short and hot summers (*98*). The choice of variables used in the causal network was based on their ecological and physical relevance. For instance, we hypothesized that the climatic water deficit, defined as the difference between actual and potential evapotranspiration, represents a crucial climatic driver influencing fire weather conditions, represented here by DC (reflecting soil drought at depths of 5 to 15 cm) in addition to the PPDSI, which captures long-term drought conditions extending for over a year. Waddington *et al.* (*99*) stressed the need to develop peat-adapted components of the Fire Weather Index (FWI) that account for peat typology and vegetation structure, both of which strongly influence peat hydrology. Building on this framework, Mortelmans *et al.* (*100*) introduced a peat-specific FWI using inputs from PEATCLSM model (*101*). Their findings showed that the peat-adapted Fine Fuel Moisture Code differed substantially from the ones of the original FWI, which shows much greater day-to-day variability. In contrast, the peat-adapted DC and DMC generally followed similar trends to those of the standard FWI system, indicating only moderate changes in their temporal dynamics. In this causality analysis, we focused primarily on the DC, and thus, the ERA5-derived dataset (*102*) was presumed reliable for this purpose. On the other hand, we assumed that minimum air temperature could play a key role in the variation of belowground peat carbon combustion. Cold nighttime air temperatures can penetrate deep soil layers in the absence of thermic insulation, potentially suppressing self-sustaining smoldering and shifting the process toward a short-lasting endothermic pyrolysis (*35*). Climate variables were derived as the summer means (including June and September to account for early and late fire season along with July and August). BA and peat fires were log-transformed due to their exponential relationship with climate drivers.

## Supplementary Materials

### This PDF file includes:

Figs. S1 to S13 Tables S1 and S2 References
