# Supplementary material for: Peat fires contribute disproportionately to Siberian fire carbon emissions
Source: Sci Adv. 2026 Mar 18;12(12):eadl2368. doi: 10.1126/sciadv.adl2368 (PMC12998507; doi:10.1126/sciadv.adl2368)
Supplement: Supplementary file 1 — Figs. S1 to S13 Tables S1 and S2 References [file sciadv.adl2368_sm.pdf]

Science Advances
AAAS

Supplementary Materials for

Peat fires contribute disproportionately to Siberian fire carbon emissions

Amin Khairoun *et al.*

Corresponding author: Amin Khairoun, amin.khairoun@uah.es

Sci. Adv. **12**, eadl2368 (2026)
DOI: 10.1126/sciadv.adl2368

This PDF file includes:

Figs. S1 to S13
Tables S1 and S2
References

Figures and Tables

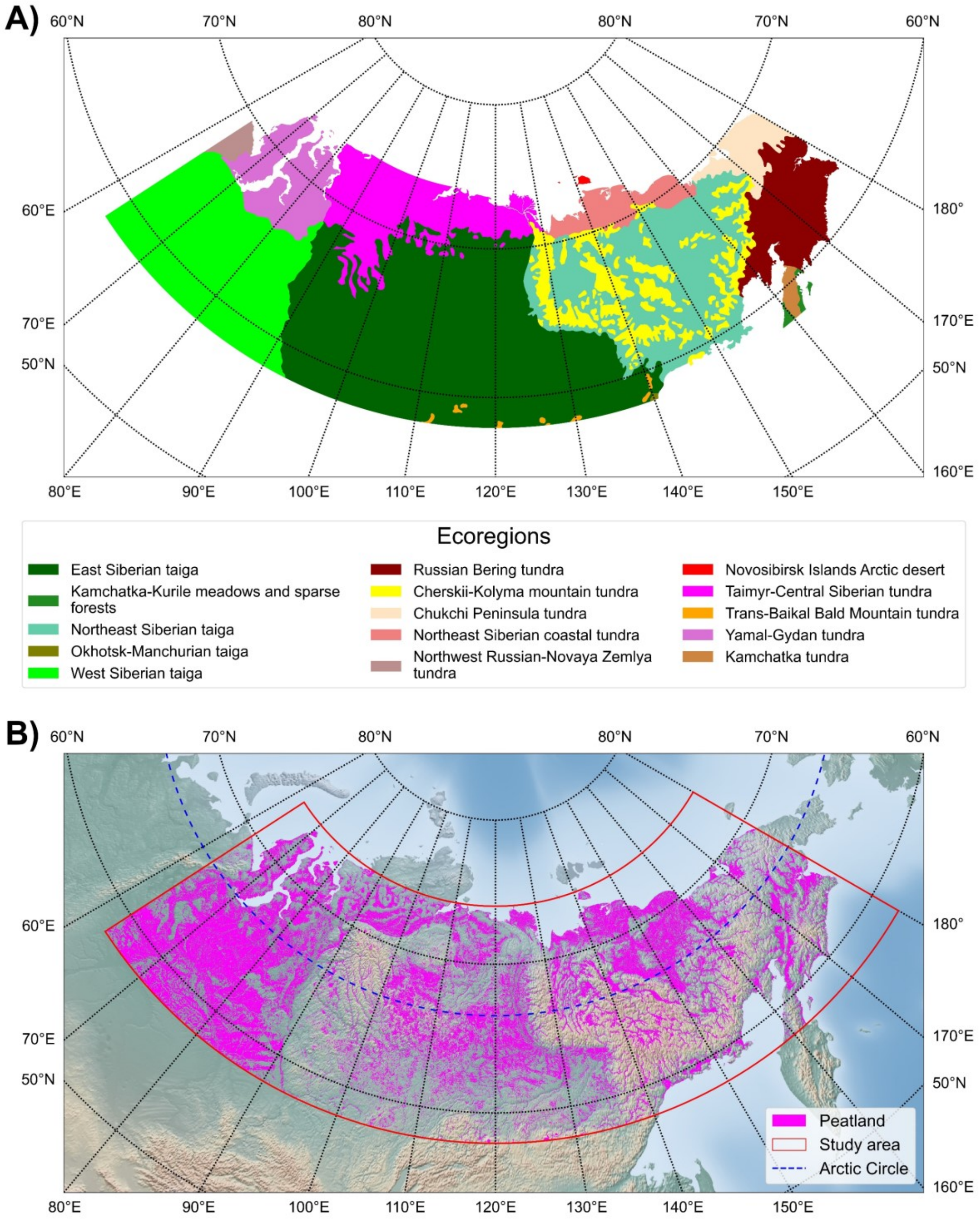

Fig. S1 | Map of Siberian ecoregions derived from Dinerstein et al. (*103*) (A) and peatland mapped area (B). We excluded the areas North of 74 degrees, typically not burning.

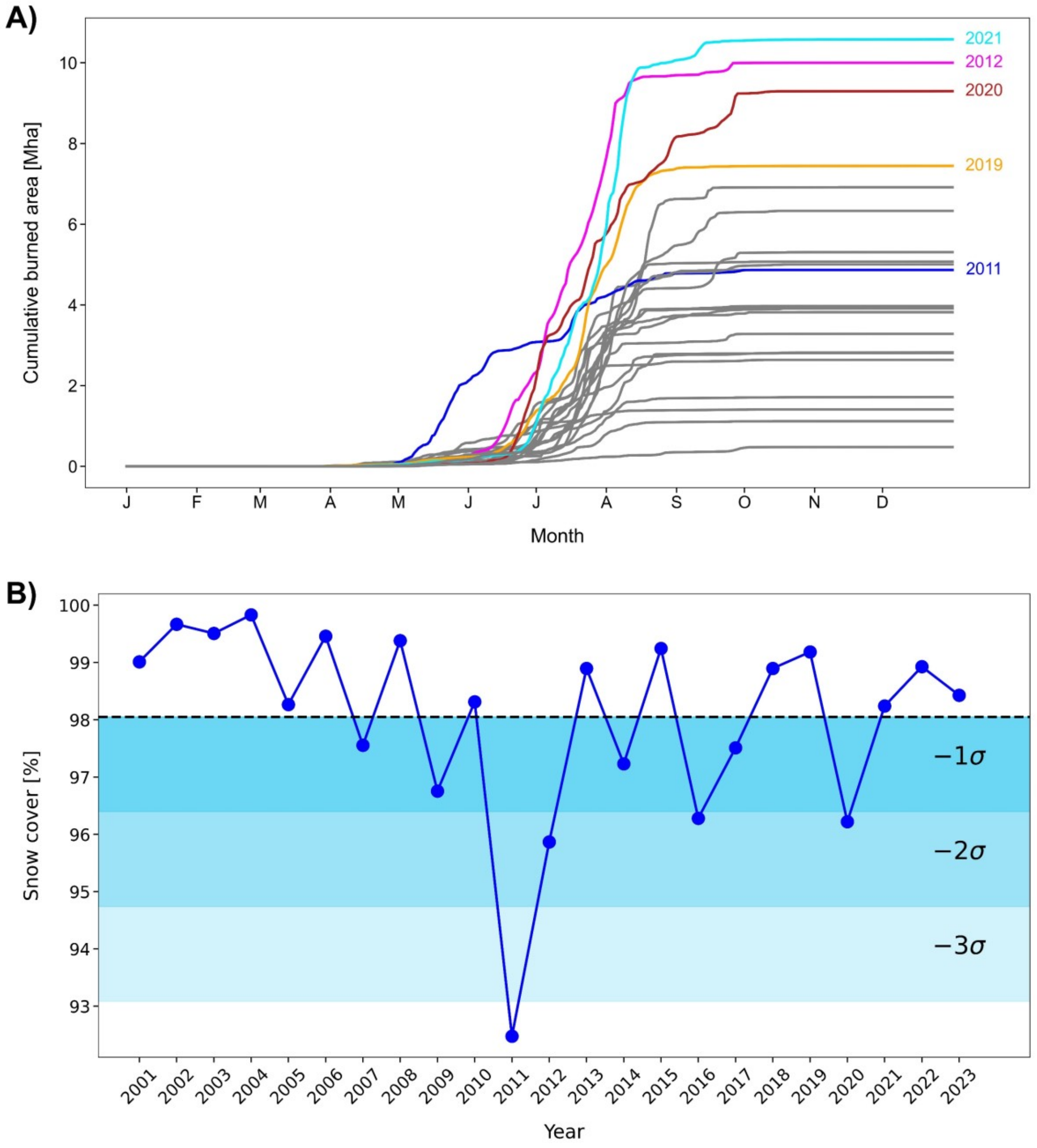

Fig. S2 | Seasonality of Siberian fires during the 2001–2023 period. A) frequency distribution of fires along fire seasons. Months are shown from January (J) to December (D); **B)** Evolution of average April snow cover percentage over years. The black dashed line designates the long-term mean of April snow cover percentage while shading indicates one, two and three one-sided standard deviations from this mean.

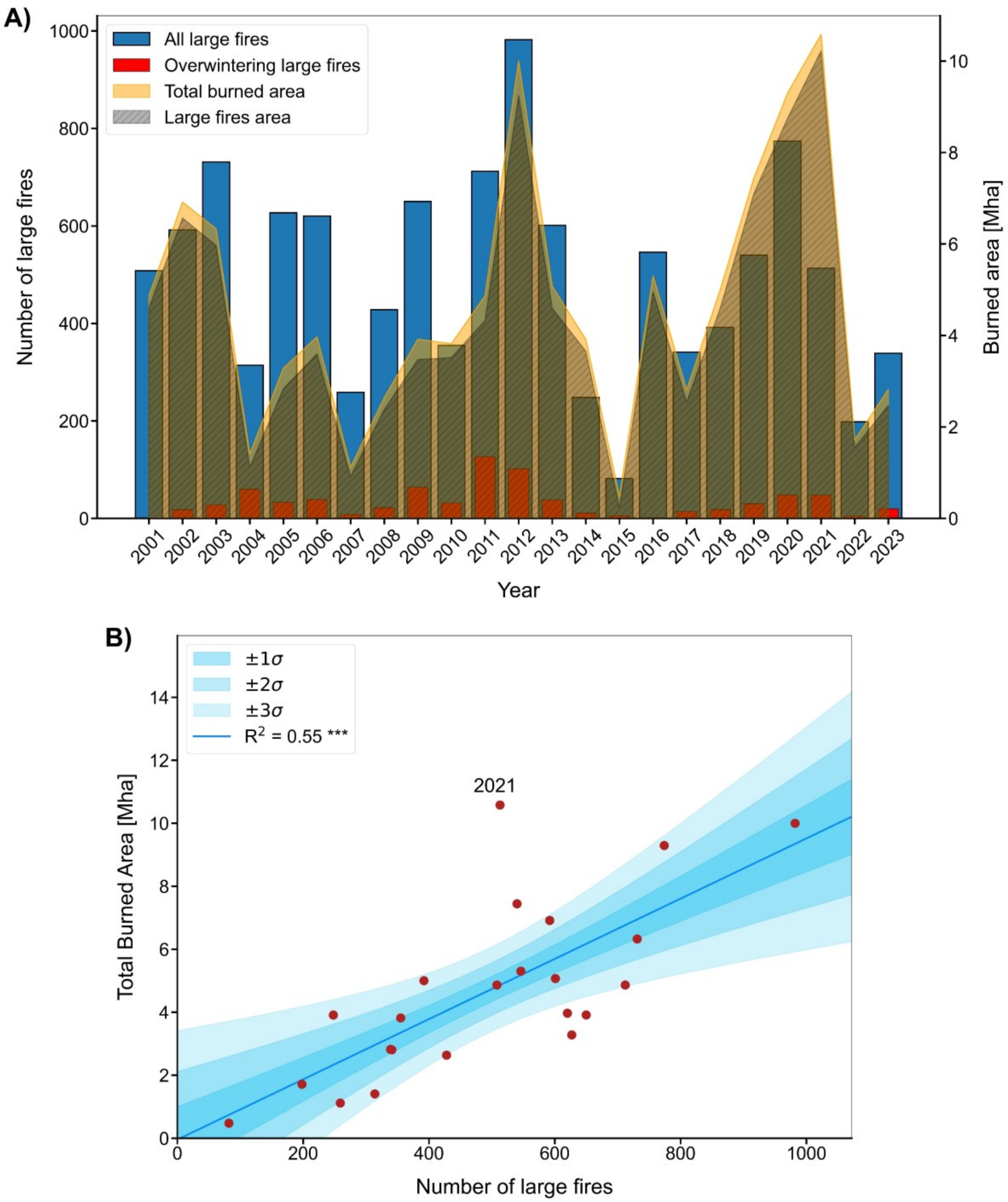

Fig. S3 | Relationship between large fires and total burned area. **A)** Yearly representation of the fraction of large fires caused by overwintering (2001 is excluded) and the fraction of total burned area resulting from large fires; **B)** Linear regression between the number of large fires and total burned area. The shading indicates one, two and three standard deviations from the regression line. Solid regression line is labelled by its corresponding coefficient of determination (***: $p < 0.001$; **: $p < 0.005$; *: $p < 0.05$).

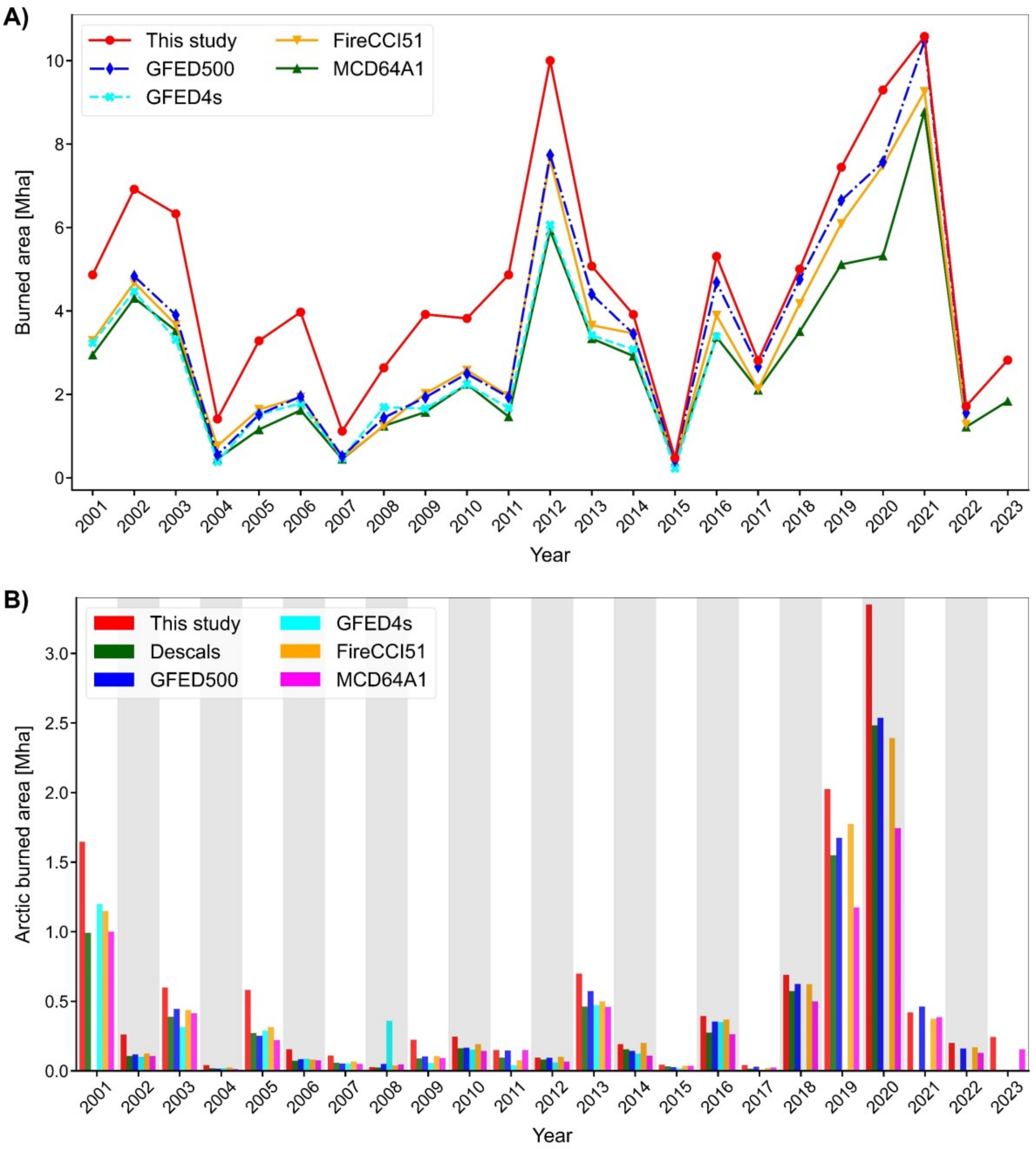

Fig. S4 | Comparison of yearly mapped burned area (This study) against other available datasets. The comparison includes conventional coarse-resolution products **(A)** in addition to a recent burned area dataset based on multiple satellite data (*33*) for the Arctic region covering a smaller subset from 72.30°E eastwards and 66.5°N northwards, to which all other products were cropped **(B)**.

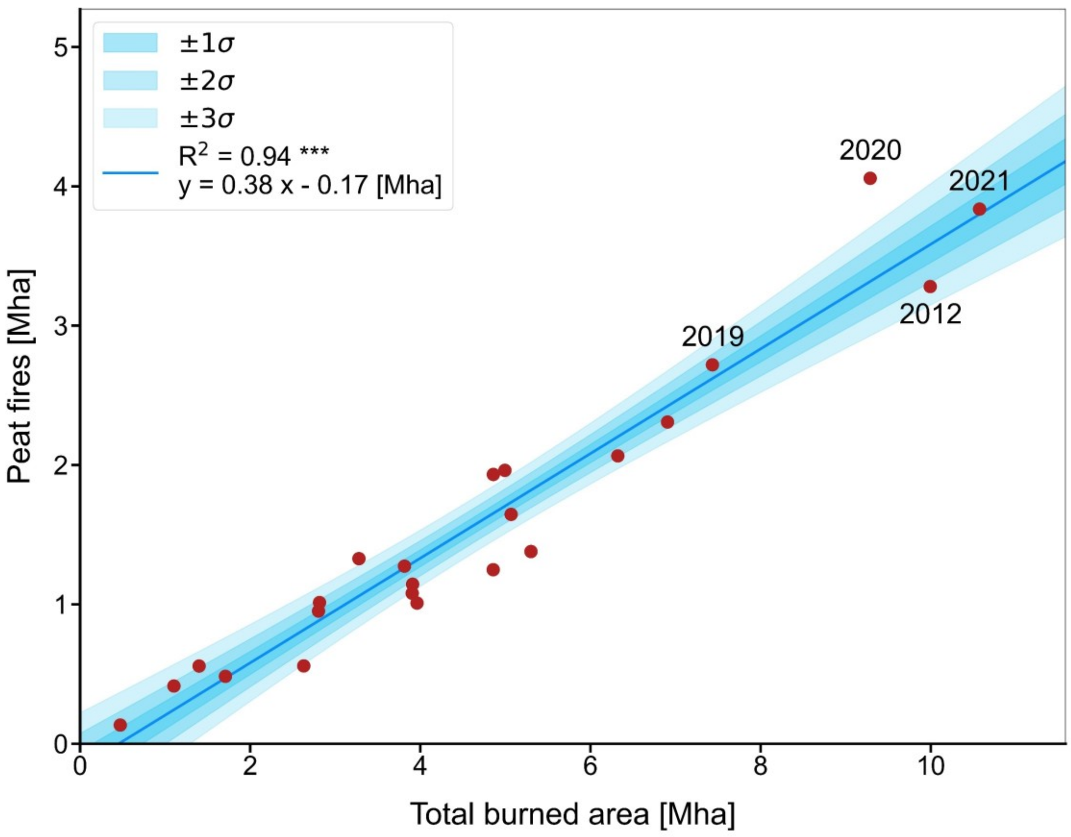

Fig. S5 | Linear regression between total annual burned area and peat fires. The shading indicates one, two and three standard deviations from the regression line. Solid regression line is labelled by its corresponding coefficient of determination (***: $p < 0.001$; **: $p < 0.005$; *: $p < 0.05$).

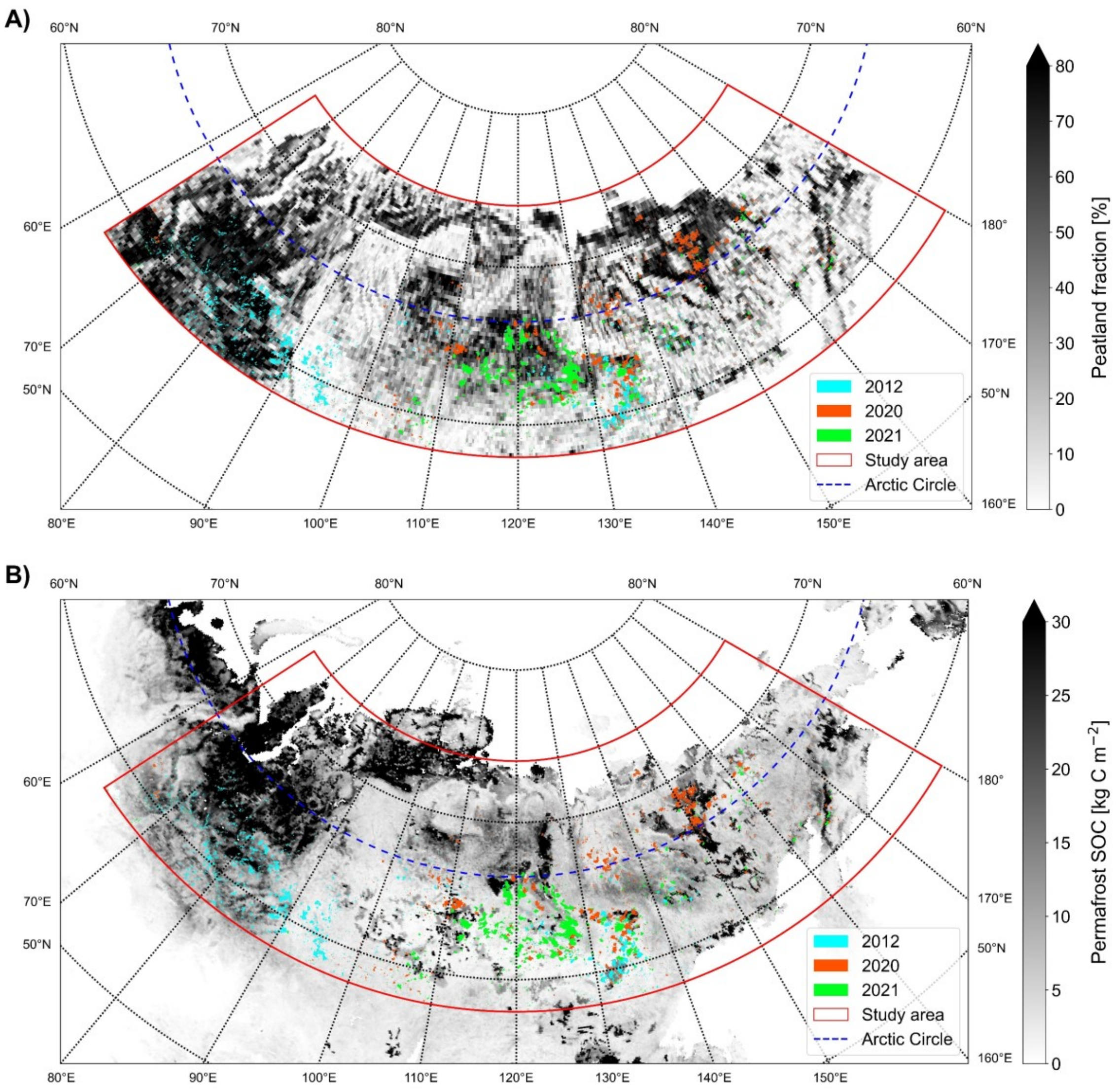

Fig. S6 | Spatial distribution of burned area in 2012, 2020, and 2021 and the corresponding soil properties. The maps show that the large fires of 2012 were concentrated in southern and southeastern Siberian taiga (near 90°E and 135°E), whereas the fires of 2021 were located in central southeastern Siberian taiga (110°E–130°E) with less peatland **(A)** and permafrost soil carbon **(B)** compared to the 2020 fires, which prevailed in northern tundra with large fractions of peatland and permafrost soil carbon.

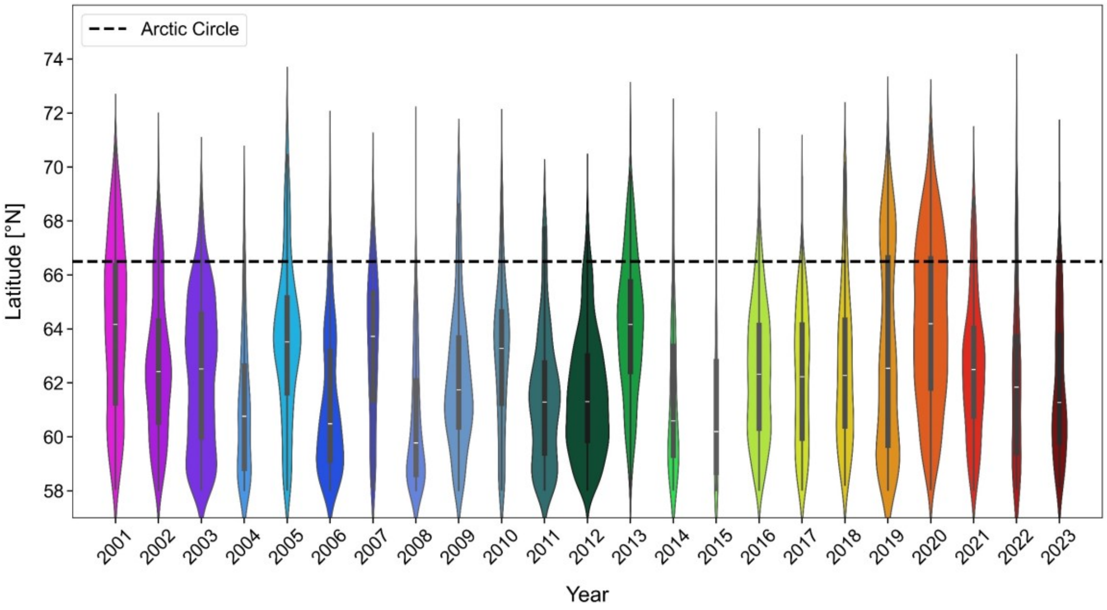

Fig. S7 | Yearly frequentist distribution of large fires by latitudes. The maximum width of each violin denotes the number of large fires (> 1000 ha) corresponding to each year.

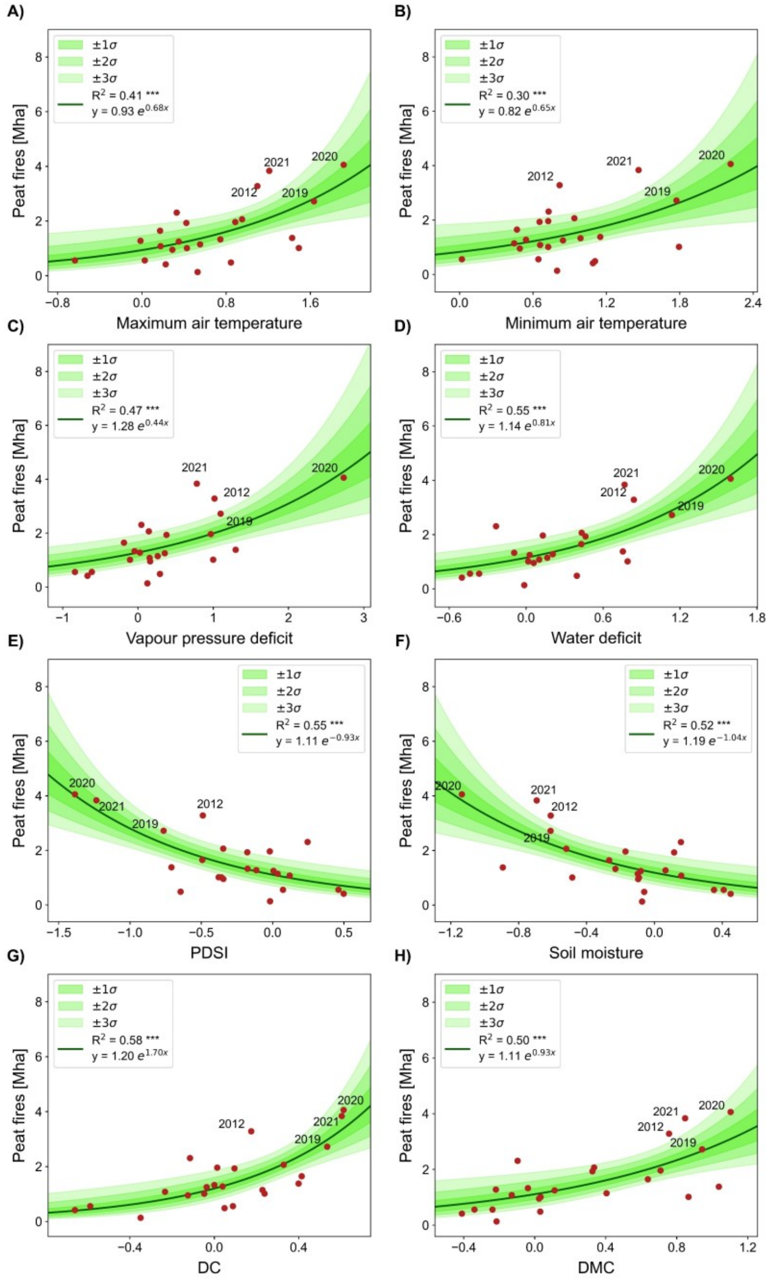

Fig. S8 | Regressions between summer climate factors (represented by anomalies from their long-term means) and peat burned area in Siberia between 2001 and 2023. The long-term climate means were calculated for the 1958–2023 period for variables derived from TerraClimate (*104*) and for the 1940–2023 period for the drought code (DC) and duff moisture code (DMC). Solid regression curves are labelled by their corresponding coefficient of determination (***: $p < 0.001$; **: $p < 0.005$; *: $p < 0.05$) and the equation of the generalized linear model. The shading indicates one, two and three standard deviations from the regression line. The anomalies were computed using z-scores.

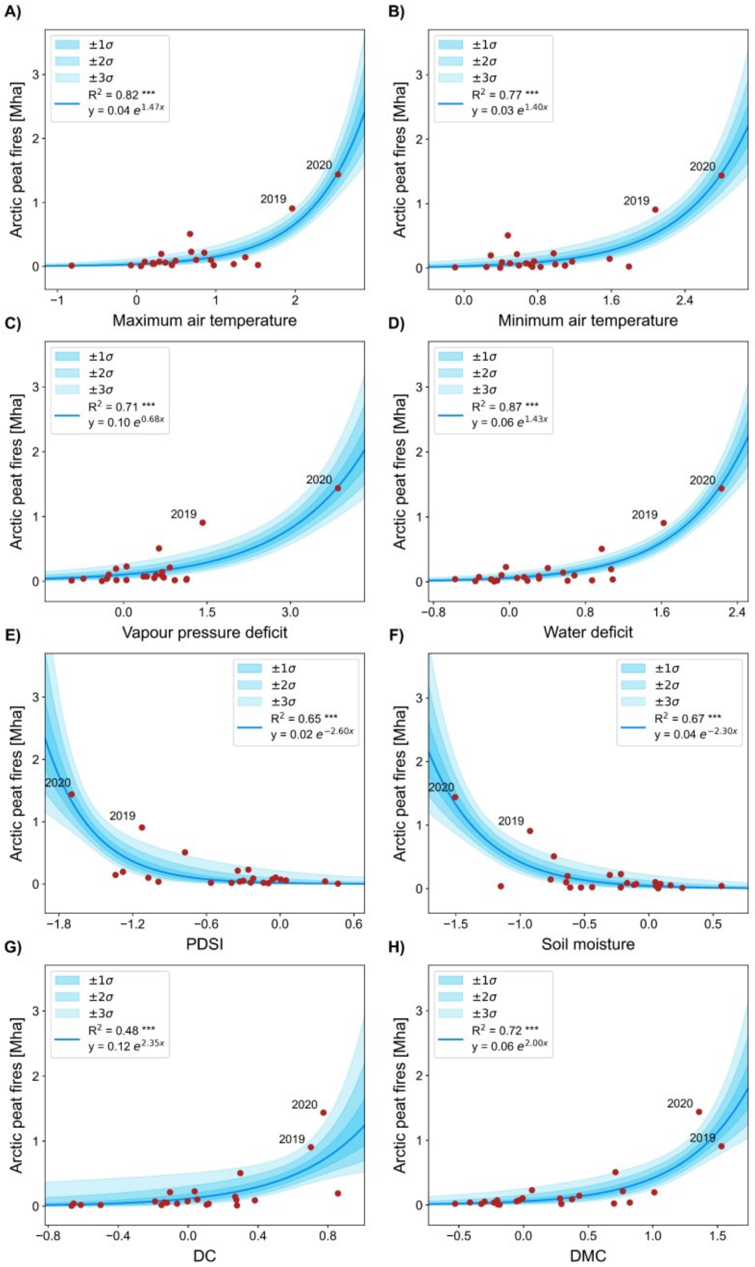

Fig. S9 | Regressions between summer climate factors (represented by anomalies from their long-term means) and peat burned area in Arctic Siberia between 2001 and 2023. The long-term climate means were calculated for the 1958–2023 period for variables derived from TerraClimate (*104*) and for the 1940–2023 period for the drought code (DC) and duff moisture code (DMC). Solid regression curves are labelled by their corresponding coefficient of determination (***: $p < 0.001$; **: $p < 0.005$; *: $p < 0.05$) and the equation of the generalized linear model. The shading indicates one, two and three standard deviations from the regression line. The anomalies were computed using z-scores.

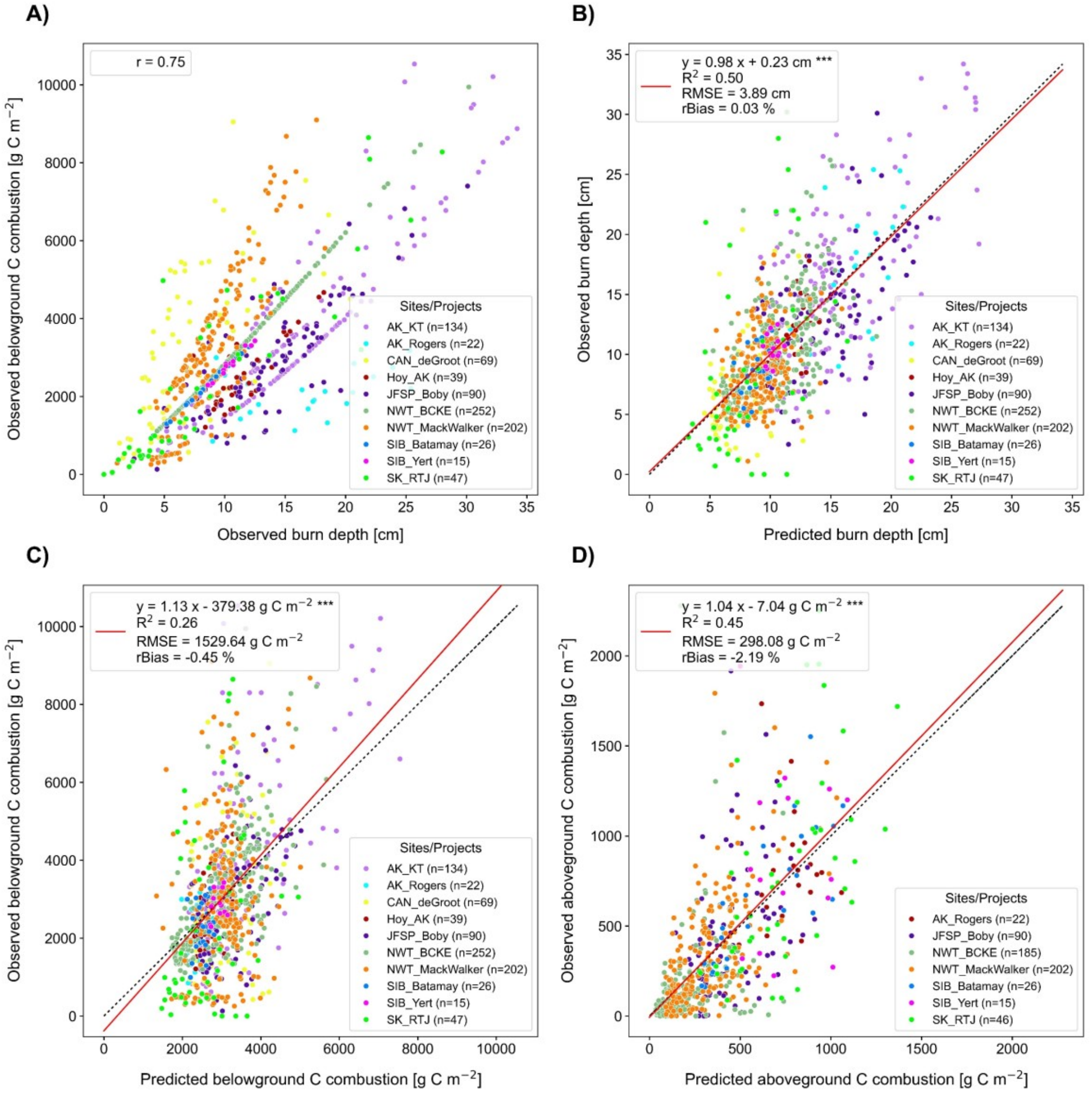

Fig. S10 | Comparison of observed and predicted burn depth and carbon combustion across different study sites/projects of origin. **(A)** Observed belowground carbon combustion as a function of observed burn depth; **(B)** Predicted burn depth versus observed burn depth; **(C)** Observed against predicted belowground carbon combustion; **(D)** Observed against predicted aboveground carbon combustion. Data points are colour-coded by study sites/projects, with a detailed legend indicating the number of observations used.

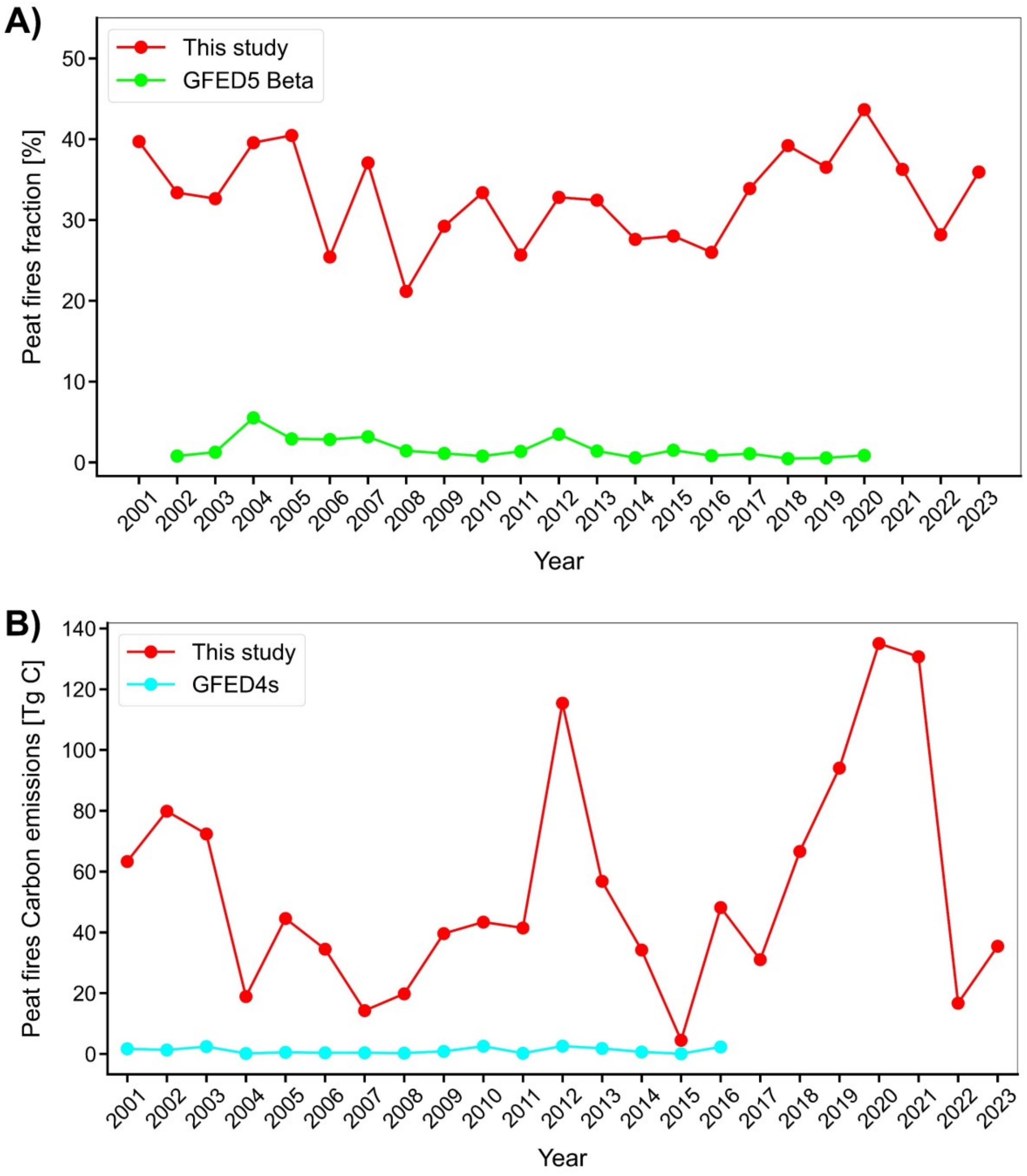

Fig. S11 | Comparison of estimated proportions of peat fires (A) and their corresponding carbon emissions (B) with the Global Fire Emissions Database (GFED) products. The 500 m GFED (*14*) does not include northern peat fires partitioning while in GFED4s (*32*), this partitioning is included only in emissions and it is supposed to be similar to GFED5 "Beta" version (*21*).

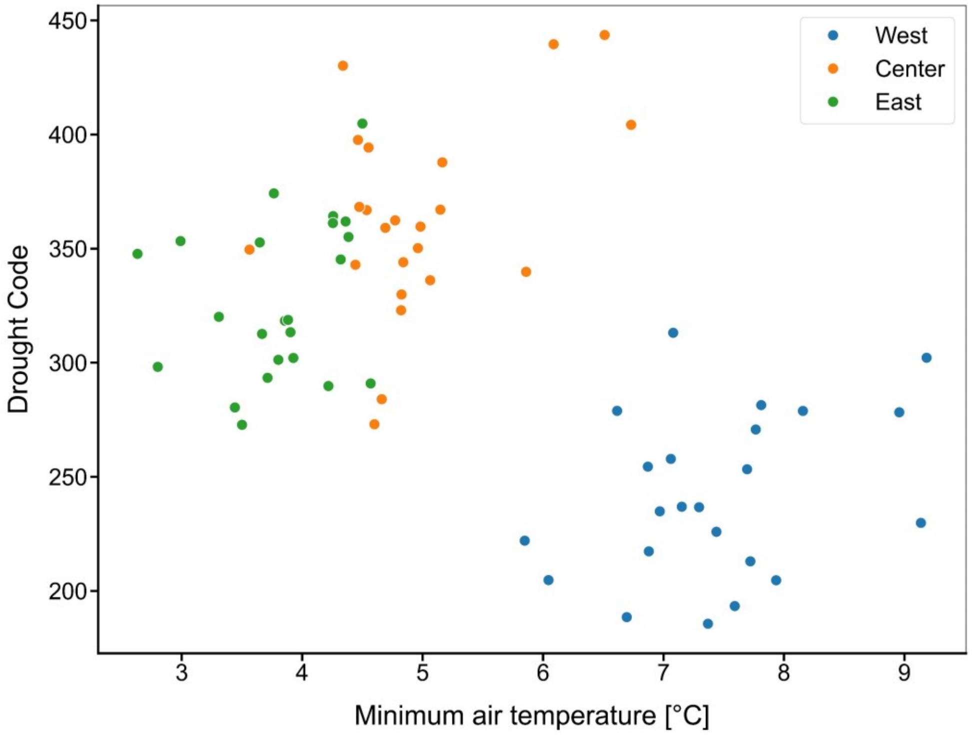

Fig. S12 | Illustration of annual summer Drought Code as a function of minimum air temperature in each subset region of Siberia.

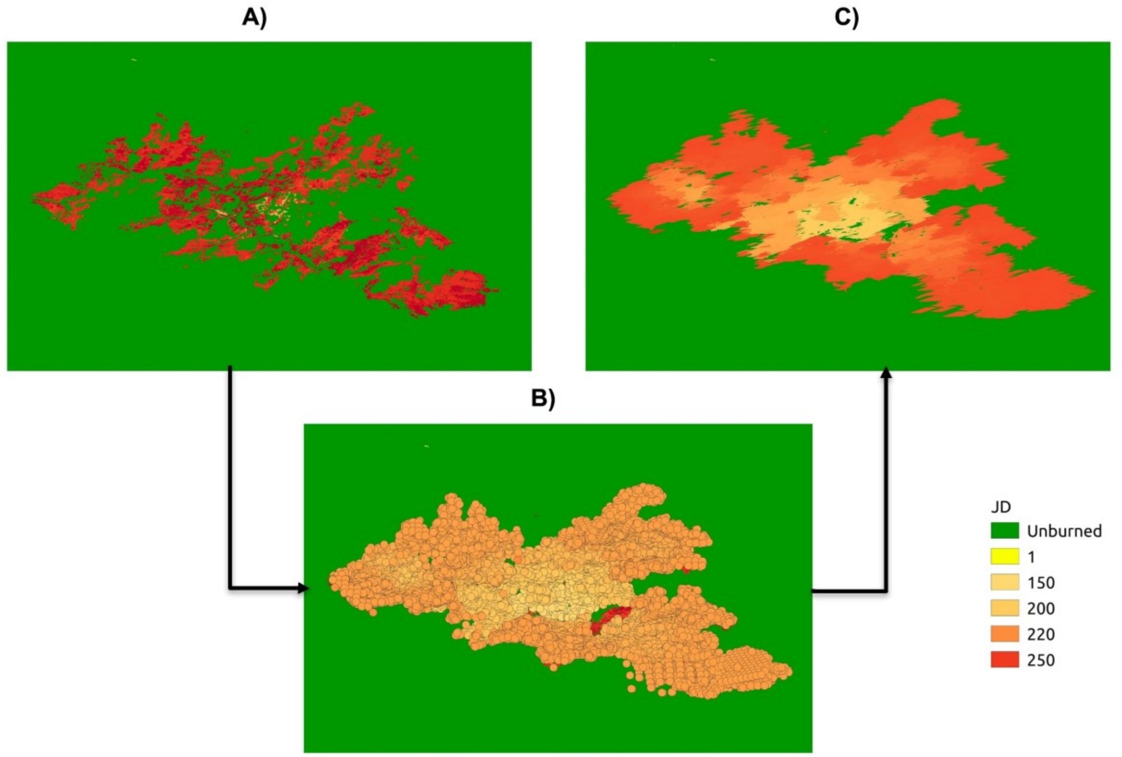

Fig. S13 | Illustration of the process of burned area mapping enhancement using active fires. The raw map of BA **(A)** was overlapped with active fires **(B)** in order to enhance the dating (Julian Day; JD) and capture undetected parts of the patch (i.e., due to clouds or low fire signal in delayed observation). Here only active fires connected to the raw BA were retained.

Table S1 | Comparison of the Landsat-derived burned area against the MCD64A1 and FireCCI51. Omission and commission errors are denoted as Oe and Ce, respectively.

Product	Oe	Ce	Dice coefficient
**This study**	**10.41**	29.46	**0.79**
**FireCCI51**	27.88	26.53	0.73
**MCD64A1**	35.86	**20.85**	0.71

Table S2 | Variables used to model burn depth and carbon combustion. Climate variables used here were calculated as the summer average (June-September), while their anomalies were computed using z-scores as $\frac{X-\mu}{\sigma}$, where X is the corresponding summer average, μ is the long-term summer average and σ its standard deviation.

Variable	Resolution	Source
**Minimum NBR** #	30 m	The BA dataset
**Minimum NBR2** #	30 m	The BA dataset
**NBR difference between pre and post-fire** #	30 m	The BA dataset
**NBR2 difference between pre and post-fire** §	30 m	The BA dataset
**Fire patch area**	30 m	The BA dataset
**Fire patch duration** §	30 m	The BA dataset
**Julian day of burn** #	30 m	The BA dataset
**Fire radiative power***	375 or 1000 m	VIIRS VNP14IMG or VJ114IMG (*105*), or MODIS MCD14ML (*84*)
**Maximum air temperature** §	(1/24°; ~ 4 km)	TerraClimate (*104*)
**Minimum air temperature** §	(1/24°; ~ 4 km)	TerraClimate (*104*)
**Vapour pressure deficit** #	(1/24°; ~ 4 km)	TerraClimate (*104*)
**Climatic water deficit** #	(1/24°; ~ 4 km)	TerraClimate (*104*)
**Soil moisture** §#	(1/24°; ~ 4 km)	TerraClimate (*104*)
**Palmer Drought Severity Index**	(1/24°; ~ 4 km)	TerraClimate (*104*)
**Downward surface shortwave radiation** §#	(1/24°; ~ 4 km)	TerraClimate (*104*)
**Maximum air temperature anomaly** §#	(1/24°; ~ 4 km)	TerraClimate (*104*)
**Minimum air temperature anomaly** §#	(1/24°; ~ 4 km)	TerraClimate (*104*)

Variable	Resolution	Source
Vapour pressure deficit anomaly [§#]	(1/24°; ~ 4 km)	TerraClimate (*104*)
Climatic water deficit anomaly [§]	(1/24°; ~ 4 km)	TerraClimate (*104*)
Soil moisture anomaly [§#]	(1/24°; ~ 4 km)	TerraClimate (*104*)
Palmer Drought Severity Index anomaly [§]	(1/24°; ~ 4 km)	TerraClimate (*104*)
Downward surface shortwave radiation anomaly [§#]	(1/24°; ~ 4 km)	TerraClimate (*104*)
Duff Moisture Code [§#]	(0.25°; ~ 25 km)	Vitolo et al. (*102*)
Fine Fuel Moisture Code [§#]	(0.25°; ~ 25 km)	Vitolo et al. (*102*)
Drought Code	(0.25°; ~ 25 km)	Vitolo et al. (*102*)
Fire Weather Index [§]	(0.25°; ~ 25 km)	Vitolo et al. (*102*)
Duff Moisture Code anomaly	(0.25°; ~ 25 km)	Vitolo et al. (*102*)
Fine Fuel Moisture Code anomaly	(0.25°; ~ 25 km)	Vitolo et al. (*102*)
Drought Code anomaly	(0.25°; ~ 25 km)	Vitolo et al. (*102*)
Fire Weather Index anomaly [‡#]	(0.25°; ~ 25 km)	Vitolo et al. (*102*)
Clay [§]	250 m	SoilGrids (*106*)
Sand [§]	250 m	SoilGrids (*106*)
Silt [§]	250 m	SoilGrids (*106*)
Land surface temperature	1000 m	MODIS MOD11A2 (*107*)
Land surface temperature anomaly [§‡#]	1000 m	MODIS MOD11A2 (*107*)
Land surface temperature persistence**	1000 m	MODIS MOD11A2 (*107*)
SOC [‡#]	0.1°; ~ 11 km	Hugelius et al. (*3*)
Bog [§‡#]	0.1°; ~ 11 km	Hugelius et al. (*3*)

Variable	Resolution	Source
**Fen** [§‡]	0.1°; ~ 11 km	Hugelius et al. (*3*)
**Elevation** [§‡]	30 m	ALOS PALSAR DEM v3.2 (*108*)
**Slope**	30 m	ALOS PALSAR DEM v3.2 (*108*)
**Aspect**	30 m	ALOS PALSAR DEM v3.2 (*108*)
**Tree cover fraction** [§‡]	250 m	MODIS MOD44B (*109*)
**Non-tree vegetation cover fraction** [§‡]	250 m	MODIS MOD44B (*109*)
**Aboveground biomass density** [#]	100 m	ESA CCI BIOMASS (*38*)

*: Land surface temperature persistence is defined here as the fraction of the summer period (June-September) in which Land surface temperature anomaly is higher than 0.5.

**: Fire radiative power was derived from active fires. From 2001 to 2011, MODIS (1000 m) was used and starting from 2012 active fires input was replaced by VIIRS VNP14IMGML (375), except for the year 2022, where gaps were observed in data collected from VIIRS sensor onboard the joint NASA/NOAA Suomi-National Polar orbiting Partnership (S-NPP) satellite and was replaced by VJ114IMG (375 m) derived from VIIRS sensor onboard NOAA-20 (JPSS-1) satellite.

[§]: Variables selected for the burn depth model.

[‡]: Variables selected for the belowground carbon combustion model.

[#]: Variables selected for the aboveground carbon combustion model.

REFERENCES

1. C. Blodau, Carbon cycling in peatlands—A review of processes and controls. *Environ. Rev.* **10**, 111–134 (2002).

2. H. Joosten, D. Clarke, *Wise Use of Mires and Peatlands: Background and Principles Including a Framework for Decision-Making* (International Mire Conservation Group and International Peat Society, 2002).

3. G. Hugelius, J. Loisel, S. Chadburn, R. B. Jackson, M. Jones, G. Macdonald, M. Marushchak, D. Olefeldt, M. Packalen, M. B. Siewert, C. Treat, M. Turetsky, C. Voigt, Z. Yu, Large stocks of peatland carbon and nitrogen are vulnerable to permafrost thaw. *Proc. Natl. Acad. Sci. U.S.A.* **117**, 20438–20446 (2020).

4. M. C. Serreze, J. A. Francis, The arctic amplification debate. *Clim. Change* **76**, 241–264 (2006).

5. M. Rantanen, A. Y. Karpechko, A. Lipponen, K. Nordling, O. Hyvärinen, K. Ruosteenoja, T. Vihma, A. Laaksonen, The Arctic has warmed nearly four times faster than the globe since 1979. *Commun. Earth Environ.* **3**, 168 (2022).

6. E. A. G. Schuur, J. Bockheim, J. G. Canadell, E. Euskirchen, C. B. Field, S. V. Goryachkin, S. Hagemann, P. Kuhry, P. M. Lafleur, H. Lee, G. Mazhitova, F. E. Nelson, A. Rinke, V. E. Romanovsky, N. Shiklomanov, C. Tarnocai, S. Venevsky, J. G. Vogel, S. A. Zimov, Vulnerability of permafrost carbon to climate change: Implications for the global carbon cycle. *Bioscience* **58**, 701–714 (2008).

7. E. A. G. Schuur, A. D. Mcguire, C. Schädel, G. Grosse, J. W. Harden, D. J. Hayes, G. Hugelius, C. D. Koven, P. Kuhry, D. M. Lawrence, S. M. Natali, D. Olefeldt, V. E. Romanovsky, K. Schaefer, M. R. Turetsky, C. C. Treat, J. E. Vonk, Climate change and the permafrost carbon feedback. *Nature* **520**, 171–179 (2015).

8. S. L. Wilkinson, R. Andersen, P. A. Moore, S. J. Davidson, G. Granath, J. M. Waddington, Wildfire and degradation accelerate northern peatland carbon release. *Nat. Clim. Change.* **13**, 456–461 (2023).

9. S. Lin, Y. Liu, X. Huang, Climate-induced Arctic-boreal peatland fire and carbon loss in the 21st century. *Sci. Total Environ.* **796**, 148924 (2021).

10. R. C. Scholten, D. Coumou, F. Luo, S. Veraverbeke, Early snowmelt and polar jet dynamics co-influence recent extreme Siberian fire seasons. *Science* **378**, 1005–1009 (2022).

11. C. M. Gibson, L. E. Chasmer, D. K. Thompson, W. L. Quinton, M. D. Flannigan, D. Olefeldt, Wildfire as a major driver of recent permafrost thaw in boreal peatlands. *Nat. Commun.* **9**, 3041 (2018).

12. S. Veraverbeke, C. J. F. Delcourt, E. Kukavskaya, M. Mack, X. Walker, T. Hessilt, B. Rogers, R. C. Scholten, Direct and longer-term carbon emissions from arctic-boreal fires: A short review of recent advances. *Curr. Opin. Environ. Sci. Health* **23**, 100277 (2021).

13. M. R. Turetsky, B. W. Abbott, M. C. Jones, K. W. Anthony, D. Olefeldt, E. A. G. Schuur, G. Grosse, P. Kuhry, G. Hugelius, C. Koven, D. M. Lawrence, C. Gibson, A. B. K. Sannel, A. D. McGuire, Carbon release through abrupt permafrost thaw. *Nat. Geosci.* **13**, 138–143 (2020).

14. D. Van Wees, G. R. Van Der Werf, J. T. Randerson, B. M. Rogers, Y. Chen, S. Veraverbeke, L. Giglio, D. C. Morton, Global biomass burning fuel consumption and emissions at 500 m spatial resolution based on the Global Fire Emissions Database (GFED). *Geosci. Model Dev.* **15**, 8411–8437 (2022).

15. J. T. Randerson, Y. Chen, G. R. Werf, B. M. Rogers, D. C. Morton, Global burned area and biomass burning emissions from small fires. *J. Geophys. Res.* **117**, G04012 (2012).

16. R. Ramo, E. Roteta, I. Bistinas, D. van Wees, A. Bastarrika, E. Chuvieco, G. R. van der Werf, African burned area and fire carbon emissions are strongly impacted by small fires undetected by coarse resolution satellite data. *Proc. Natl. Acad. Sci. U.S.A.* **118**, e2011160118 (2021).

17. B. Zheng, P. Ciais, F. Chevallier, E. Chuvieco, Y. Chen, H. Yang, Increasing forest fire emissions despite the decline in global burned area. *Sci. Adv.* **7**, eabh2646 (2021).

18. A. Khairoun, F. Mouillot, W. Chen, P. Ciais, E. Chuvieco, Coarse-resolution burned area datasets severely underestimate fire-related forest loss. *Sci. Total Environ.* **920**, 170599 (2024).

19. M. R. Turetsky, B. Benscoter, S. Page, G. Rein, G. R. Van Der Werf, A. Watts, Global vulnerability of peatlands to fire and carbon loss. *Nat. Geosci.* **8**, 11–14 (2015).

20. X. Pan, C. Ichoku, M. Chin, H. Bian, A. Darmenov, P. Colarco, L. Ellison, T. Kucsera, A. Da Silva, J. Wang, T. Oda, G. Cui, Six global biomass burning emission datasets: Intercomparison and application in one global aerosol model. *Atmos. Chem. Phys.* **20**, 969–994 (2020).

21. Y. Chen, J. Hall, D. Van Wees, N. Andela, S. Hantson, L. Giglio, G. R. Van Der Werf, D. C. Morton, J. T. Randerson, Multi-decadal trends and variability in burned area from the fifth version of the Global Fire Emissions Database (GFED5). *Earth Syst. Sci. Data* **15**, 5227–5259 (2023).

22. A. A. Clelland, G. J. Marshall, R. Baxter, S. Potter, A. C. Talucci, J. M. Rady, H. Genet, B. M. Rogers, S. M. Natali, Annual and seasonal patterns of burned area products in arctic-boreal North America and Russia for 2001–2020. *Remote Sens.* **16**, 3306 (2024).

23. I. R. Van Der Velde, G. R. Van Der Werf, D. Van Wees, N. A. J. Schutgens, R. Vernooij, S. Houweling, E. Tonucci, E. Chuvieco, J. T. Randerson, M. M. Frey, T. Borsdorff, I. Aben, Small fires, big impact: Evaluating fire emission estimates in Southern Africa using new satellite imagery of burned area and carbon monoxide. *Geophys. Res. Lett.* **51**, e2023GL106122 (2024).

24. S. S. Rabin, J. R. Melton, G. Lasslop, D. Bachelet, M. Forrest, S. Hantson, J. O. Kaplan, F. Li, S. Mangeon, D. S. Ward, C. Yue, V. K. Arora, T. Hickler, S. Kloster, W. Knorr, L. Nieradzik, A. Spessa, G. A. Folberth, T. Sheehan, A. Voulgarakis, D. I. Kelley, I. C. Prentice, S. Sitch, S. Harrison, A. Arneth, The Fire Modeling Intercomparison Project (FireMIP), phase 1: Experimental and analytical protocols with detailed model descriptions. *Geosci. Model Dev.* **10**, 1175–1197 (2017).

25. J. Xu, P. J. Morris, J. Liu, J. Holden, PEATMAP: Refining estimates of global peatland distribution based on a meta-analysis. *CATENA* **160**, 134–140 (2018).

26. J. R. Melton, E. Chan, K. Millard, M. Fortier, R. S. Winton, J. M. Martín-López, H. Cadillo-Quiroz, D. Kidd, L. V. Verchot, A map of global peatland extent created using machine learning (Peat-ML). *Geosci. Model Dev.* **15**, 4709–4738 (2022).

27. United Nations Environment Programme, "Global Peatlands Assessment: The State of the World's Peatlands—Evidence for Action toward the Conservation, Restoration, and Sustainable Management of Peatlands" (UNEP, 2022); https://wedocs.unep.org/20.500.11822/41222.

28. D. Olefeldt, M. Hovemyr, M. A. Kuhn, D. Bastviken, T. J. Bohn, J. Connolly, P. Crill, E. S. Euskirchen, S. A. Finkelstein, H. Genet, G. Grosse, L. I. Harris, L. Heffernan, M. Helbig, G. Hugelius, R. Hutchins, S. Juutinen, M. J. Lara, A. Malhotra, K. Manies, A. D. Mcguire, S. M. Natali, J. A. O'Donnell, F.-J. W. Parmentier, A. Räsänen, C. Schädel, O. Sonnentag, M. Strack, S. E. Tank, C. Treat, R. K. Varner, T. Virtanen, R. K. Warren, J. D. Watts, The Boreal–Arctic Wetland and Lake Dataset (BAWLD). *Earth Syst. Sci. Data* **13**, 5127–5149 (2021).

29. J. L. Mccarty, T. E. L. Smith, M. R. Turetsky, Arctic fires re-emerging. *Nat. Geosci.* **13**, 658–660 (2020).

30. R. C. Scholten, R. Jandt, E. A. Miller, B. M. Rogers, S. Veraverbeke, Overwintering fires in boreal forests. *Nature* **593**, 399–404 (2021).

31. M. Franquesa, J. Lizundia-loiola, S. V. Stehman, E. Chuvieco, Using long temporal reference units to assess the spatial accuracy of global satellite-derived burned area products. *Remote Sens. Environ.* **269**, –112823 (2022).

32. G. R. van der Werf, J. T. Randerson, L. Giglio, T. T. van Leeuwen, Y. Chen, B. M. Rogers, M. Mu, M. J. E. van Marle, D. C. Morton, G. J. Collatz, R. J. Yokelson, P. S. Kasibhatla, Global fire emissions estimates during 1997–2016. *Earth Syst. Sci. Data* **9**, 697–720 (2017).

33. A. Descals, D. L. A. Gaveau, A. Verger, D. Sheil, D. Naito, J. Peñuelas, Unprecedented fire activity above the Arctic Circle linked to rising temperatures. *Science* **378**, 532–537 (2022).

34. B. W. Benscoter, D. K. Thompson, J. M. Waddington, M. D. Flannigan, B. M. Wotton, W. J. De Groot, M. R. Turetsky, Interactive effects of vegetation, soil moisture and bulk density on depth of burning of thick organic soils. *Int. J. Wildland Fire* **20**, 418–429 (2011).

35. G. Rein, X. Huang, Smouldering wildfires in peatlands, forests and the arctic: Challenges and perspectives. *Curr. Opin. Environ. Sci. Health* **24**, 100296 (2021).

36. N. Kettridge, M. R. Turetsky, J. H. Sherwood, D. K. Thompson, C. A. Miller, B. W. Benscoter, M. D. Flannigan, B. M. Wotton, J. M. Waddington, Moderate drop in water table increases peatland vulnerability to post-fire regime shift. *Sci. Rep.* **5**, 8063 (2015).

37. T. Chen, C. Guestrin, XGBoost: A scalable tree boosting system, in *Proceedings of the 22nd ACM SIGKDD International Conference on Knowledge Discovery and Data Mining* (ACM, 2016), pp. 785–794.

38. M. Santoro, O. Cartus, ESA Biomass Climate Change Initiative (Biomass_cci): Global datasets of forest above-ground biomass for the years 2010, 2017, 2018, 2019 and 2020, v4 (NERC EDS Centre for Environmental Data Analysis, 2023).

39. C. J. F. Delcourt, B. M. Rogers, L. Akhmetzyanov, B. Izbicki, R. C. Scholten, T. A. Shestakova, D. Van Wees, M. C. Mack, U. Sass-Klaassen, S. Veraverbeke, Carbon emissions from fires in eastern siberian larch forests. *Glob. Chang. Biol.* **31**, e70247 (2025).

40. M. Friedl, D. Sulla-Menashe, MCD12Q1 MODIS/Terra+Aqua Land Cover Type Yearly L3 Global 500m SIN Grid V006 (NASA EOSDIS Land Processes Distributed Active Archive Center, 2019).

41. A. M. Virkkala, B. M. Rogers, J. D. Watts, K. A. Arndt, S. Potter, I. Wargowsky, E. A. G. Schuur, C. R. See, M. Mauritz, J. Boike, M. S. Bret-Harte, E. J. Burke, A. Burrell, N. Chae, A. Chatterjee, F. Chevallier, T. R. Christensen, R. Commane, H. Dolman, C. W. Edgar, B. Elberling, C. A. Emmerton, E. S. Euskirchen, L. Feng, M. Göckede, A. Grelle, M. Helbig, D.

Holl, J. Järveoja, S. V. Karsanaev, H. Kobayashi, L. Kutzbach, J. Liu, I. T. Luijkx, E. López-Blanco, K. Lunneberg, I. Mammarella, M. E. Marushchak, M. Mastepanov, Y. Matsuura, T. C. Maximov, L. Merbold, G. Meyer, M. B. Nilsson, Y. Niwa, W. Oechel, P. I. Palmer, S.-J. Park, F.-J. W. Parmentier, M. Peichl, W. Peters, R. Petrov, W. Quinton, C. Rödenbeck, T. Sachs, C. Schulze, O. Sonnentag, V. L. St. Louis, E.-S. Tuittila, M. Ueyama, A. Varlagin, D. Zona, S. M. Natali, Wildfires offset the increasing but spatially heterogeneous Arctic–boreal CO_2 uptake. *Nat. Clim. Change.* **15**, 188–195 (2025).

42. Z. Zhu, C. E. Woodcock, Continuous change detection and classification of land cover using all available Landsat data. *Remote Sens. Environ.* **144**, 152–171 (2014).

43. M. Hethcoat, P. Jain, M.-A. Parisien, R. Skakun, L. Rogic, E. Whitman, Unrecorded tundra fires in Canada, 1986–2022. *Remote Sens.* **16**, 230 (2024).

44. W. Xu, R. C. Scholten, T. D. Hessilt, Y. Liu, S. Veraverbeke, Overwintering fires rising in eastern Siberia. *Environ. Res. Lett.* **17**, 045005 (2022).

45. D. Olefeldt, M. R. Turetsky, P. M. Crill, A. D. McGuire, Environmental and physical controls on northern terrestrial methane emissions across permafrost zones. *Glob. Chang. Biol.* **19**, 589–603 (2013).

46. A. V. Kirdyanov, M. Saurer, R. Siegwolf, A. A. Knorre, A. S. Prokushkin, O. V. Churakova (Sidorova), M. V. Fonti, U. Büntgen, Long-term ecological consequences of forest fires in the continuous permafrost zone of Siberia. *Environ. Res. Lett.* **15**, 034061 (2020).

47. X. J. Walker, J. L. Baltzer, S. G. Cumming, N. J. Day, C. Ebert, S. Goetz, J. F. Johnstone, S. Potter, B. M. Rogers, E. A. G. Schuur, M. R. Turetsky, M. C. Mack, Increasing wildfires threaten historic carbon sink of boreal forest soils. *Nature* **572**, 520–523 (2019).

48. A. C. Talucci, M. M. Loranty, J. E. Holloway, B. M. Rogers, H. D. Alexander, N. Baillargeon, J. L. Baltzer, L. T. Berner, A. Breen, L. Brodt, B. Buma, J. Dean, C. J. F. Delcourt, L. R. Diaz, C. M. Dieleman, T. A. Douglas, G. V. Frost, B. V. Gaglioti, R. E. Hewitt, T. Hollingsworth, M. T. Jorgenson, M. J. Lara, R. A. Loehman, M. C. Mack, K. L. Manies, C. Minions, S. M. Natali, J. A. O'Donnell, D. Olefeldt, A. K. Paulson, A. V. Rocha, L. B.

Saperstein, T. A. Shestakova, S. Sistla, O. Sizov, A. Soromotin, M. R. Turetsky, S. Veraverbeke, M. A. Walvoord, Permafrost–wildfire interactions: Active layer thickness estimates for paired burned and unburned sites in northern high latitudes. *Earth Syst. Sci. Data* **17**, 2887–2909 (2025).

49. B. D. Amiro, A. L. Orchansky, A. G. Barr, T. A. Black, S. D. Chambers, F. S. Chapin, M. L. Gouldenf, M. Litvakg, H. P. Liu, J. H. McCaughey, A. McMillan, J. T. Randerson, The effect of post-fire stand age on the boreal forest energy balance. *Agric. For. Meteorol.* **140**, 41–50 (2006).

50. J. P. Fisher, C. Estop-Aragonés, A. Thierry, D. J. Charman, S. A. Wolfe, I. P. Hartley, J. B. Murton, M. Williams, G. K. Phoenix, The influence of vegetation and soil characteristics on active-layer thickness of permafrost soils in boreal forest. *Glob. Chang. Biol.* **22**, 3127–3140 (2016).

51. N. Zhang, T. Yasunari, T. Ohta, Dynamics of the larch taiga–permafrost coupled system in Siberia under climate change. *Environ. Res. Lett.* **6**, 024003 (2011).

52. N. Kettridge, D. K. Thompson, L. Bombonato, M. R. Turetsky, B. W. Benscoter, J. M. Waddington, The ecohydrology of forested peatlands: Simulating the effects of tree shading on moss evaporation and species composition. *J. Geophys. Res.* **118**, 422–435 (2013).

53. I. W. Kim, A. Timmermann, J.-E. Kim, K. B. Rodgers, S.-S. Lee, H. Lee, W. R. Wieder, Abrupt increase in Arctic-Subarctic wildfires caused by future permafrost thaw. *Nat. Commun.* **15**, 7868 (2024).

54. E. Roteta, A. Bastarrika, M. Franquesa, E. Chuvieco, Landsat and sentinel-2 based burned area mapping tools in google earth engine. *Remote Sens.* **13**, 816 (2021).

55. P. Defourny, C. Lamarche, S. Bontemps, T. De Maet, E. Van Bogaert, I. Moreau, C. Brockmann, M. Boettcher, G. Kirches, J. Wevers, M. Santoro, F. Ramoino, O. Arino, "Land Cover Climate Change Initiative—Product User Guide v2. Issue 2.0" (European Space Agency (ESA), 2017); http://maps.elie.ucl.ac.be/CCI/viewer/download/ESACCI-LC-Ph2-PUGv2_2.0.pdf.

56. J. Lizundia-Loiola, M. Franquesa, A. Khairoun, E. Chuvieco, Global burned area mapping from Sentinel-3 Synergy and VIIRS active fires. *Remote Sens. Environ.* **282**, 113298 (2022).

57. A. Khairoun, J. Lizundia-Loiola, E. Chuvieco, M. L. Pettinari, T. Storm, M. Boettcher, G. Kirches, "ESA CCI ECV Fire Disturbance: D2.2 Algorithm Theoretical Basis Document-SYN, version 1.1" (European Space Agency (ESA), 2023); https://climate.esa.int/en/projects/fire/key-documents/.

58. Z. Zhu, S. Wang, C. E. Woodcock, Improvement and expansion of the Fmask algorithm: Cloud, cloud shadow, and snow detection for Landsats 4–7, 8, and Sentinel 2 images. *Remote Sens. Environ.* **159**, 269–277 (2015).

59. J. C. Storey, M. J. Choate, Landsat-5 bumper-mode geometric correction. *IEEE Trans. Geosci. Remote Sens.* **42**, 2695–2703 (2004).

60. A. H. Pickens, M. C. Hansen, M. Hancher, S. V. Stehman, A. Tyukavina, P. Potapov, B. Marroquin, Z. Sherani, Mapping and sampling to characterize global inland water dynamics from 1999 to 2018 with full Landsat time-series. *Remote Sens. Environ.* **243**, 111792 (2020).

61. D. Oom, P. C. Silva, I. Bistinas, J. M. C. Pereira, Highlighting biome-specific sensitivity of fire size distributions to time-gap parameter using a new algorithm for fire event individuation. *Remote Sens.* **8**, 663 (2016).

62. P. Laurent, F. Mouillot, C. Yue, P. Ciais, M. V. Moreno, J. M. P. Nogueira, FRY, a global database of fire patch functional traits derived from space-borne burned area products. *Sci. Data* **5**, 180132 (2018).

63. N. Andela, D. C. Morton, L. Giglio, R. Paugam, Y. Chen, S. Hantson, G. R. van der Werf, J. T. Randerson, The global fire atlas of individual fire size, duration, speed, and direction. *Earth Syst. Sci. Data* **11**, 529–552 (2019).

64. S. Archibald, R. J. Scholes, D. P. Roy, G. Roberts, L. Boschetti, Southern African fire regimes as revealed by remote sensing. *Int. J. Wildland Fire* **19**, 861–878 (2010).

65. Y. Fan, H. Li, G. Miguez-Macho, Global patterns of groundwater table depth. *Science* **339**, 940–943 (2013).

66. H. Joosten, *The Global Peatland CO_2 Picture: Peatland status and emissions in all countries of the world* (Wetlands International, 2009), pp. 35.

67. C. Zaccone, G. Rein, V. D'Orazio, R. M. Hadden, C. M. Belcher, T. M. Miano, Smouldering fire signatures in peat and their implications for palaeoenvironmental reconstructions. *Geochim. Cosmochim. Acta* **137**, 134–146 (2014).

68. M. Franquesa, M. K. Vanderhoof, D. Stavrakoudis, I. Z. Gitas, E. Roteta, M. Padilla, E. Chuvieco, Development of a standard database of reference sites for validating global burned area products. *Earth Syst. Sci. Data* **12**, 3229–3246 (2020).

69. M. Franquesa Fuentetaja, M. K. Vanderhoof, D. Stavrakoudis, I. Gitas, E. Roteta, M. Padilla, E. Chuvieco, BARD: a global and regional validation burned area database (e-cienciaDatos, 2020).

70. L. Giglio, L. Boschetti, D. P. Roy, M. L. Humber, C. O. Justice, The collection 6 MODIS burned area mapping algorithm and product. *Remote Sens. Environ.* **217**, 72–85 (2018).

71. J. Lizundia-Loiola, G. Otón, R. Ramo, E. Chuvieco, A spatio-temporal active-fire clustering approach for global burned area mapping at 250 m from MODIS data. *Remote Sens. Environ.* **236**, 111493 (2020).

72. W. Seiler, P. J. Crutzen, Estimates of gross and net fluxes of carbon between the biosphere and the atmosphere from biomass burning. *Clim. Change* **2**, 207–247 (1980).

73. X. J. Walker, J. L. Baltzer, L. L. Bourgeau-Chavez, N. J. Day, W. J. De Groot, C. Dieleman, E. E. Hoy, J. F. Johnstone, E. S. Kane, M. A. Parisien, S. Potter, B. M. Rogers, M. R. Turetsky, S. Veraverbeke, E. Whitman, M. C. Mack, ABoVE: Synthesis of Burned and Unburned Forest Site Data, AK and Canada, 1983-2016 (ORNL DAAC, 2020).

74. C. J. F. Delcourt, A. Combee, B. Izbicki, M. C. Mack, T. Maximov, R. Petrov, B. M. Rogers, R. C. Scholten, T. A. Shestakova, D. van Wees, S. Veraverbeke, Evaluating the differenced

normalized burn ratio for assessing fire severity using sentinel-2 imagery in Northeast Siberian larch forests. *Remote Sens.* **13**, 2311 (2021).

75. M. R. Turetsky, E. S. Kane, J. W. Harden, R. D. Ottmar, K. L. Manies, E. Hoy, E. S. Kasischke, Recent acceleration of biomass burning and carbon losses in Alaskan forests and peatlands. *Nat. Geosci.* **4**, 27–31 (2010).

76. S. Potter, S. Cooperdock, S. Veraverbeke, X. Walker, M. C. Mack, S. J. Goetz, J. Baltzer, L. Bourgeau-Chavez, A. Burrell, C. Dieleman, N. French, S. Hantson, E. E. Hoy, L. Jenkins, J. F. Johnstone, E. S. Kane, S. M. Natali, J. T. Randerson, M. R. Turetsky, E. Whitman, E. Wiggins, B. M. Rogers, Burned area and carbon emissions across northwestern boreal North America from 2001–2019. *Biogeosciences* **20**, 2785–2804 (2023).

77. J. Bergstra, B. Komer, C. Eliasmith, D. Yamins, D. D. Cox, Hyperopt: A Python library for model selection and hyperparameter optimization. *Comput. Sci. Discov.* **8**, 014008 (2015).

78. J. Bergstra, R. Bardenet, Y. Bengio, B. Kégl, Algorithms for hyper-parameter optimization. *Adv. Neural Inf. Process. Syst.* **24**, (2011).

79. B. Matérn, *Spatial Variation—Stochastic models and their application ·to some problems in forest surveys and other sampling investigations* (Springer, 1960).

80. M. L. Stein, *Interpolation of Spatial Data* (Springer Series in Statistics, 1999).

81. C. E. Rasmussen, C. K. I. Williams, *Gaussian Processes for Machine Learning* (MIT Press, 2005).

82. S. Sarkka, A. Solin, J. Hartikainen, Spatiotemporal learning via infinite-dimensional bayesian filtering and smoothing: A look at gaussian process regression through kalman filtering. *IEEE Signal Process. Mag.* **30**, 51–61 (2013).

83. P. Drineas, M. W. Mahoney, On the nyström method for approximating a gram matrix for improved kernel-based learning. *J. Mach. Learn. Res.* **6**, 2153–2175 (2005).

84. L. Giglio, W. Schroeder, C. O. Justice, The collection 6 MODIS active fire detection algorithm and fire products. *Remote Sens. Environ.* **178**, 31–41 (2016).

85. C. B. Field, J. T. Randerson, C. M. Malmström, Global net primary production: Combining ecology and remote sensing. *Remote Sens. Environ.* **51**, 74–88 (1995).

86. C. S. Potter, J. T. Randerson, C. B. Field, P. A. Matson, P. M. Vitousek, H. A. Mooney, S. A. Klooster, Terrestrial ecosystem production: A process model based on global satellite and surface data. *Glob. Biogeochem. Cycles* **7**, 811–841 (1993).

87. M. J. Wooster, Small-scale experimental testing of fire radiative energy for quantifying mass combusted in natural vegetation fires. *Geophys. Res. Lett.* **29**, 23-1–23-4 (2002).

88. M. Wooster, B. Zhukov, D. Oertel, Fire radiative energy for quantitative study of biomass burning: Derivation from the BIRD experimental satellite and comparison to MODIS fire products. *Remote Sens. Environ.* **86**, 83–107 (2003).

89. J. W. Kaiser, A. Heil, M. O. Andreae, A. Benedetti, N. Chubarova, L. Jones, J. J. Morcrette, M. Razinger, M. G. Schultz, M. Suttie, G. R. van der Werf, Biomass burning emissions estimated with a global fire assimilation system based on observed fire radiative power. *Biogeosciences* **9**, 527–554 (2012).

90. A. S. d. S. Darmenov, M. Arlindo, "The Quick Fire Emissions Dataset (QFED): Documentation of Versions 2.1, 2.2 and 2.4" (NASA/TM-2015-104606 2015); https://gmao.gsfc.nasa.gov/pubs/docs/Darmenov796.pdf.

91. C. Wiedinmyer, S. K. Akagi, R. J. Yokelson, L. K. Emmons, J. A. Al-Saadi, J. J. Orlando, A. J. Soja, The Fire INventory from NCAR (FINN): A high resolution global model to estimate the emissions from open burning. *Geosci. Model Dev.* **4**, 625–641 (2011).

92. C. Wiedinmyer, Y. Kimura, E. C. Mcdonald-Buller, L. K. Emmons, R. R. Buchholz, W. Tang, K. Seto, M. B. Joseph, K. C. Barsanti, A. G. Carlton, R. Yokelson, The Fire Inventory from NCAR version 2.5: An updated global fire emissions model for climate and chemistry applications. *Geosci. Model Dev.* **16**, 3873–3891 (2023).

93. B. Zheng, P. Ciais, F. Chevallier, H. Yang, J. G. Canadell, Y. Chen, I. R. van der Velde, I. Aben, E. Chuvieco, S. J. Davis, M. Deeter, C. Hong, Y. Kong, H. Li, H. Li, X. Lin, K. He, Q. Zhang, Record-high CO_2 emissions from boreal fires in 2021. *Science* **379**, 912–917 (2023).

94. E. B. Wiggins, A. Andrews, C. Sweeney, J. B. Miller, C. E. Miller, S. Veraverbeke, R. Commane, S. Wofsy, J. M. Henderson, J. T. Randerson, Boreal forest fire CO_2 and CH4 emission factors derived from tower observations in Alaska during the extreme fire season of 2015. *Atmos. Chem. Phys.* **21**, 8557–8574 (2021).

95. X. J. Walker, B. M. Rogers, S. Veraverbeke, J. F. Johnstone, J. L. Baltzer, K. Barrett, L. Bourgeau-Chavez, N. J. Day, W. J. De Groot, C. M. Dieleman, S. Goetz, E. Hoy, L. K. Jenkins, E. S. Kane, M.-A. Parisien, S. Potter, E. A. G. Schuur, M. Turetsky, E. Whitman, M. C. Mack, Fuel availability not fire weather controls boreal wildfire severity and carbon emissions. *Nat. Clim. Change.* **10**, 1130–1136 (2020).

96. J. S. Lefcheck, piecewiseSEM: Piecewise structural equation modelling in R for ecology, evolution, and systematics. *Methods Ecol. Evol.* **7**, 573–579 (2016).

97. K. E. Frey, L. C. Smith, Recent temperature and precipitation increases in West Siberia and their association with the Arctic Oscillation. *Polar Res.* **22**, 287–300 (2003).

98. N. M. Tchebakova, E. Parfenova, A. J. Soja, The effects of climate, permafrost and fire on vegetation change in Siberia in a changing climate. *Environ. Res. Lett.* **4**, 045013 (2009).

99. J. M. Waddington, D. K. Thompson, M. Wotton, W. L. Quinton, M. D. Flannigan, B. W. Benscoter, S. A. Baisley, M. R. Turetsky, Examining the utility of the Canadian forest fire weather index system in boreal peatlands. *Can. J. For. Res.* **42**, 47–58 (2012).

100. J. Mortelmans, A. Felsberg, G. J. M. De Lannoy, S. Veraverbeke, R. D. Field, N. Andela, M. Bechtold, Improving the fire weather index system for peatlands using peat-specific hydrological input data. *Nat. Hazards Earth Syst. Sci.* **24**, 445–464 (2024).

101. M. Bechtold, G. J. M. De Lannoy, R. D. Koster, R. H. Reichle, S. P. Mahanama, W. Bleuten, M. A. Bourgault, C. Brümmer, I. Burdun, A. R. Desai, K. Devito, T. Grünwald, M.

Grygoruk, E. R. Humphreys, J. Klatt, J. Kurbatova, A. Lohila, T. M. Munir, M. B. Nilsson, J. S. Price, PEAT-CLSM: A specific treatment of peatland hydrology in the NASA catchment land surface model. *J. Adv. Model. Earth Syst.* **11**, 2130–2162 (2019).

102. C. Vitolo, F. Di Giuseppe, C. Barnard, R. Coughlan, J. San-Miguel-Ayanz, G. Libertá, B. Krzeminski, ERA5-based global meteorological wildfire danger maps. *Sci. Data* **7**, 216 (2020).

103. E. Dinerstein, D. Olson, A. Joshi, C. Vynne, N. D. Burgess, E. Wikramanayake, N. Hahn, S. Palminteri, P. Hedao, R. Noss, M. Hansen, H. Locke, E. C. Ellis, B. Jones, C. V. Barber, R. Hayes, C. Kormos, V. Martin, E. Crist, W. Sechrest, L. Price, J. E. M. Baillie, D. Weeden, K. Suckling, C. Davis, N. Sizer, R. Moore, D. Thau, T. Birch, P. Potapov, S. Turubanova, A. Tyukavina, N. de Souza, L. Pintea, J. C. Brito, O. A. Llewellyn, A. G. Miller, A. Patzelt, S. A. Ghazanfar, J. Timberlake, H. Kloser, Y. Shennan-Farpon, R. Kindt, J. B. Lilleso, P. van Breugel, L. Graudal, M. Voge, K. F. Al-Shammari, M. Saleem, An ecoregion-based approach to protecting half the terrestrial realm. *Bioscience* **67**, 534–545 (2017).

104. J. T. Abatzoglou, S. Z. Dobrowski, S. A. Parks, K. C. Hegewisch, TerraClimate, a high-resolution global dataset of monthly climate and climatic water balance from 1958–2015. *Sci. Data* **5**, 170191 (2018).

105. W. Schroeder, P. Oliva, L. Giglio, I. A. Csiszar, The new VIIRS 375 m active fire detection data product: Algorithm description and initial assessment. *Remote Sens. Environ.* **143**, 85–96 (2014).

106. L. Poggio, L. M. de Sousa, N. H. Batjes, G. B. M. Heuvelink, B. Kempen, E. Ribeiro, D. Rossiter, SoilGrids 2.0: Producing soil information for the globe with quantified spatial uncertainty. *Soil* **7**, 217–240 (2021).

107. Z. Wan, S. Hook, G. Hulley, MODIS/Terra Land Surface Temperature/Emissivity 8-Day L3 Global 1km SIN Grid V061 (NASA EOSDIS Land Processes Distributed Active Archive Center, 2021).

108. T. Tadono, H. Ishida, F. Oda, S. Naito, K. Minakawa, H. Iwamoto, Precise global DEM generation by ALOS PRISM. *ISPRS Ann. Photogramm. Remote Sens. Spatial Inf.* **II-4**, 71–76 (2014).

109. C. DiMiceli, R. Sohlberg, J. Townshend, MODIS/Terra Vegetation Continuous Fields Yearly L3 Global 250m SIN Grid V061 (NASA EOSDIS Land Processes Distributed Active Archive Center, 2022).
